# Selective LRRK2 kinase inhibition reduces phosphorylation of endogenous Rab10 and Rab12 in human peripheral mononuclear blood cells

**DOI:** 10.1038/s41598-017-10501-z

**Published:** 2017-08-31

**Authors:** Kenneth Thirstrup, Justus C. Dächsel, Felix S. Oppermann, Douglas S. Williamson, Garrick P. Smith, Karina Fog, Kenneth V. Christensen

**Affiliations:** 1 Neurodegeneration and Biologics, H. Lundbeck A/S, Ottiliavej 9, Valby, DK-2500 Denmark; 2 Discovery Chemistry, H. Lundbeck A/S, Ottiliavej 9, DK-2500 Valby, Denmark; 3 Vernalis (R&D) Ltd, Granta Park, Great Abington, Cambridge, CB21 6GB United Kingdom; 4 Evotec (München) GmbH, Am Klopferspitz 19a, 82152 Martinsried, Germany

## Abstract

Genetic variation in the leucine-rich repeat kinase 2 (*LRRK2*) gene is associated with risk of familial and sporadic Parkinson’s disease (PD). To support clinical development of LRRK2 inhibitors as disease-modifying treatment in PD biomarkers for kinase activity, target engagement and kinase inhibition are prerequisite tools. In a combined proteomics and phosphoproteomics study on human peripheral mononuclear blood cells (PBMCs) treated with the LRRK2 inhibitor Lu AF58786 a number of putative biomarkers were identified. Among the phospho-site hits were known LRRK2 sites as well as two phospho-sites on human Rab10 and Rab12. LRRK2 dependent phosphorylation of human Rab10 and human Rab12 at positions Thr73 and Ser106, respectively, was confirmed in HEK293 and, more importantly, Rab10-pThr73 inhibition was validated in immune stimulated human PBMCs using two distinct LRRK2 inhibitors. In addition, in non-stimulated human PBMCs acute inhibition of LRRK2 with two distinct LRRK2 inhibitor compounds reduced Rab10-Thr73 phosphorylation in a concentration-dependent manner with apparent IC_50_’s equivalent to IC_50_’s on LRRK2-pSer935. The identification of Rab10 phosphorylated at Thr73 as a LRRK2 inhibition marker in human PBMCs strongly support inclusion of assays quantifying Rab10-pThr73 levels in upcoming clinical trials evaluating LRRK2 kinase inhibition as a disease-modifying treatment principle in PD.

## Introduction

Rare missense mutations in *LRRK2* cause clinical and pathological phenotypes of Parkinson’s disease (PD) that are undistinguishable from idiopathic PD^1–5^. Recent studies have shown that also common *LRRK2* genetic variants are associated with risk of developing late onset PD^6–11^. Together, this suggests that LRRK2 signaling pathways may be central to the processes underlying both *LRRK2* familial and sporadic late onset PD. One way to investigate the underlying role of *LRRK2* in sporadic PD is to identify specific fingerprints reflecting LRRK2 kinase function and validate these in samples from patients suffering from idiopathic PD. In the past, efforts to perform such studies have been restricted by a limited understanding of LRRK2 function, its associated cellular signaling pathways as well as lack of good and selective pharmacological tools. Also, the predominant number of studies attempting to identify LRRK2 specific read-outs has been based on non-mammalian as well as non-physiological systems including various levels of exogenous over-expression of LRRK2 and other genetic manipulations^12–16^.

In rodent and man LRRK2 is predominantly expressed in brain, spleen, lung, kidney and immune cells^17–22^. Besides association to Parkinson’s disease *LRRK2* genetic variation has also been associated with risk of multiple systems atrophy (MSA)^23^ and outside the CNS, *LRRK2* has been associated with several diseases of the immune system^24–28^. Thus, we hypothesize that LRRK2 cellular mechanisms and signaling pathways are either conserved or exhibit high similarity between the central nervous system (CNS) and peripheral target tissues such as lung, kidney and immune cells. In support, LRRK2 phosphorylation at Ser910, Ser935, Ser955 and Ser973 used to assess LRRK2 enzymatic function is detected in all target tissues^29–33^. In rodents, phosphorylation of Ser910 and Ser935 is observed in brain, kidney, spleen and blood cells and phosphorylation levels correlate well between different tissues supporting the hypothesis that cellular mechanisms regulating LRRK2 function are conserved. Further, genetic ablation and pharmalogical inhibition of LRRK2 kinase function in rodents and non-human primates have identified LRRK2 kinase activity-dependent kidney and lung phenotypes^34–38^. Another example of a conserved LRRK2 fingerprint is the LRRK2-Ser1292 autophosphorylation site^39, 40^ and more recent, in rodent transgenic models several small Rab GTPases have been confirmed as *in vitro* and *in vivo* substrates of LRRK2^41^. These observations are of particular interest since in PD GWAS *LRRK2* has previously been shown to genetically interact with *PARK16* which is encoded by the *RAB7L1* gene^10, 42^. LRRK2 and phosphorylated LRRK2 have been observed in human blood, urine and CSF from healthy controls and PD patients^43–45^. In preclinical models LRRK2 inhibition with potent and selective LRRK2 kinase inhibitors has consistently been shown to reduce LRRK2 phosphorylation, autophosphorylation and now also substrate phosphorylation^33, 37, 41, 46–50^. Collectively, LRRK2 appears to play an important physiological role outside the brain and its cellular fingerprints e.g. LRRK2 phosphorylation, LRRK2 auto phosphorylation and substrate phosphorylation seem well conserved between CNS and non-CNS cells. Still what has not been described are effects of LRRK2 inhibition on LRRK2 autophosphorylation and LRRK2 substrate phosphorylation in human primary cells expressing endogenous levels of LRRK2. In peripheral blood mononuclear cells (PBMCs) LRRK2 mRNA and protein expression can be induced by treatment with reagents known to initiate an immune response^51–53^. Thus, by applying PBMCs as a human LRRK2 model system, the goal of the underlying study was to identify novel LRRK2 kinase activity dependent substrates.

## Results

### LRRK2 inhibition in immune stimulated human PBMCs

**Figure 1:**
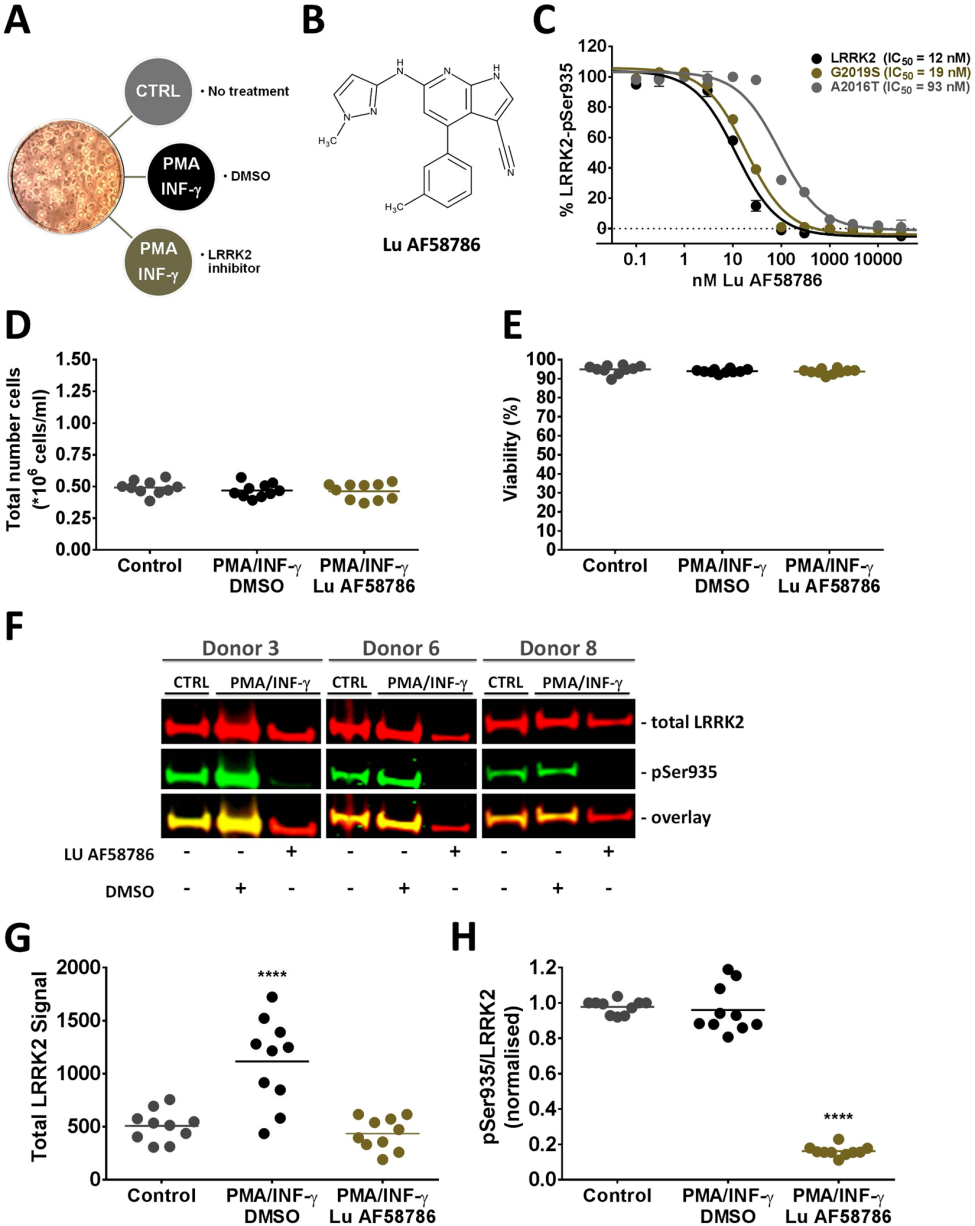
LRRK2 expression and inhibition in cultured human PBMCs immune stimulated with PMA and interferon-**γ**. (**A**) Schematic representation of the three different treatment conditions. (**B**) Chemical structure of the LRRK2 inhibitor Lu AF58786. (**C**) Determination of LRRK2, G2019S and A2016T IC_50_ values using the Odyssey CLx 96-well ICW assay (mean IC_50_, n = 3 experiments). (**D**) Total PBMC yield obtained from human healthy donors after 3 days *in vitro* (3 DIV) (n = 10 donors; 3 different conditions). (**E**) % viability of human PBMCs (n = 10 donors; 3 different conditions) after 3 DIV. (**F**) Odyssey CLx scan image of Western Blot used for estimation of LRRK2 protein levels (total LRRK2) and LRRK2 phosphorylation (pSer935). The concentration of Lu AF58786 was 100 nM. Full-length blots are presented in Supplementary Figure 1. (**G**) Quantification of total LRRK2 levels (raw signal). (**H**) Relative LRRK2-pSer935 phosphorylation (pSer935/total LRRK2). Data was analyzed by one-way ANOVA with Dunnett’s multiple comparisons test. Data presented as means ± SEM; ****p < 0.0001 vs. control. CTRL, control; PMA, phorbol 12-myristate 13-acetate; INF-γ, interferon-γ; DMSO, dimethyl sulfoxide.

LRRK2 protein expression in human blood cells is increased upon treatment with PMA and INF-γ^51^. Presumably, this induction occurs under physiological conditions e.g. as part of the inflammatory response to viral infections^54^. Hypothesizing that LRRK2 signaling pathways are conserved between the CNS and the periphery we intended to identify novel LRRK2 kinase activity dependent substrates in PBMCs from healthy human individuals. To reduce variability between individual donors and to increase the likelihood of identifying LRRK2 kinase activity dependent substrates immune stimulated PBMCs were pursued in mass spectrometry studies. Firstly, studies were performed to ensure quality of PBMC sampling and culturing, that LRRK2 protein expression could be increased and that LRRK2 kinase inhibition could be obtained after LRRK2 inhibitor treatment. For that purpose three experimental conditions were assessed (Fig. 1A). In the control condition human PBMCs were isolated and cultured for three days. An increase in LRRK2 protein expression was stimulated by treating the cultured PBMCs with an immune stimulating cocktail consisting of 400 nM PMA and 100 ng/ml INF-γ and finally, LRRK2 kinase activity in the cultured and immune stimulated PBMCs was inhibited by treatment with 100 nM Lu AF58786, a potent and selective LRRK2 inhibitor (Fig. 1B). Lu AF58786 was originally discovered as part of a joint Lundbeck-Vernalis chemistry program aimed at identifying inhibitor compounds targeting the LRRK2 kinase domain^55^. The discovery series from which Lu AF58786 was identified will be disclosed in a later publication (Williamson *et al*., in preparation). In transiently transfected HEK293 cells the IC_50_ for Lu AF58786 on LRRK2 WT, the overactive variant G2019S and the inhibition resistant mutant A2016T^56^ were 12 nM, 19 nM and 93 nM, respectively (Fig. 1C). The cell-based inhibition data confirms that Lu AF58786 is a potent inhibitor of LRRK2 and G2019S that also engages alanine at position 2016 in LRRK2. The kinase selectivity of Lu AF58786 was assessed in human PBMCs using the ActivX KiNativ assay. Encouragingly, Lu AF58786 gave 93% inhibition on LRRK2 at 1 µM and 83% at 100 nM (Supplementary File 1). There was no inhibition of other kinases at 100 nM supporting that Lu AF58786 selectively inhibits LRRK2 in human PBMCs.

Human PBMC’s isolated from 10 healthy human subjects were cultured and treated according to the three above-mentioned conditions (Fig. 1A and Material and Methods). Following 3 days of culturing and immune stimulation as well as 24hr compound treatment PBMCs were harvested and yield as well as viability was assessed. There were no significant differences in PBMC yield or viability between subjects or treatments (Fig. 1D,E). The average yield was ~0.4 × 10^6^ cells pr. sample and average viability was above 94%. All PBMC isolations were subjected to SDS-PAGE and subsequent Western Blot analysis using antibodies recognizing total LRRK2 and LRRK2-pSer935 to assess both the induction of LRRK2 expression as well as the inhibition of LRRK2-Ser935 phosphorylation (Fig. 1F–H). Western Blot based analyses showed LRRK2 expression was significantly induced in immune stimulated samples when compared to the non-stimulated condition (Fig. 1F, G). In terms of LRRK2 inhibition, Western blot analysis of samples treated with 100 nM of the selective LRRK2 inhibitor Lu AF58786 showed inhibition of LRRK2-Ser935 phosphorylation (Fig. 1F). Of notice, LRRK2 inhibition also significantly lowers the total LRRK2 protein levels (Fig. 1G﻿). Quantification of the relative LRRK2-Ser935 phosphorylation showed full inhibition for all samples processed (Fig. 1H). Collectively, the human PBMC optimization experiments showed robust induction of total LRRK2 and LRRK2-pSer935 expression as well as full LRRK2 kinase inhibition upon LRRK2 kinase inhibition (Fig. 1G,H).

### LRRK2 kinase activity dependent proteomic and phosphoproteomic studies

**Figure 2:**
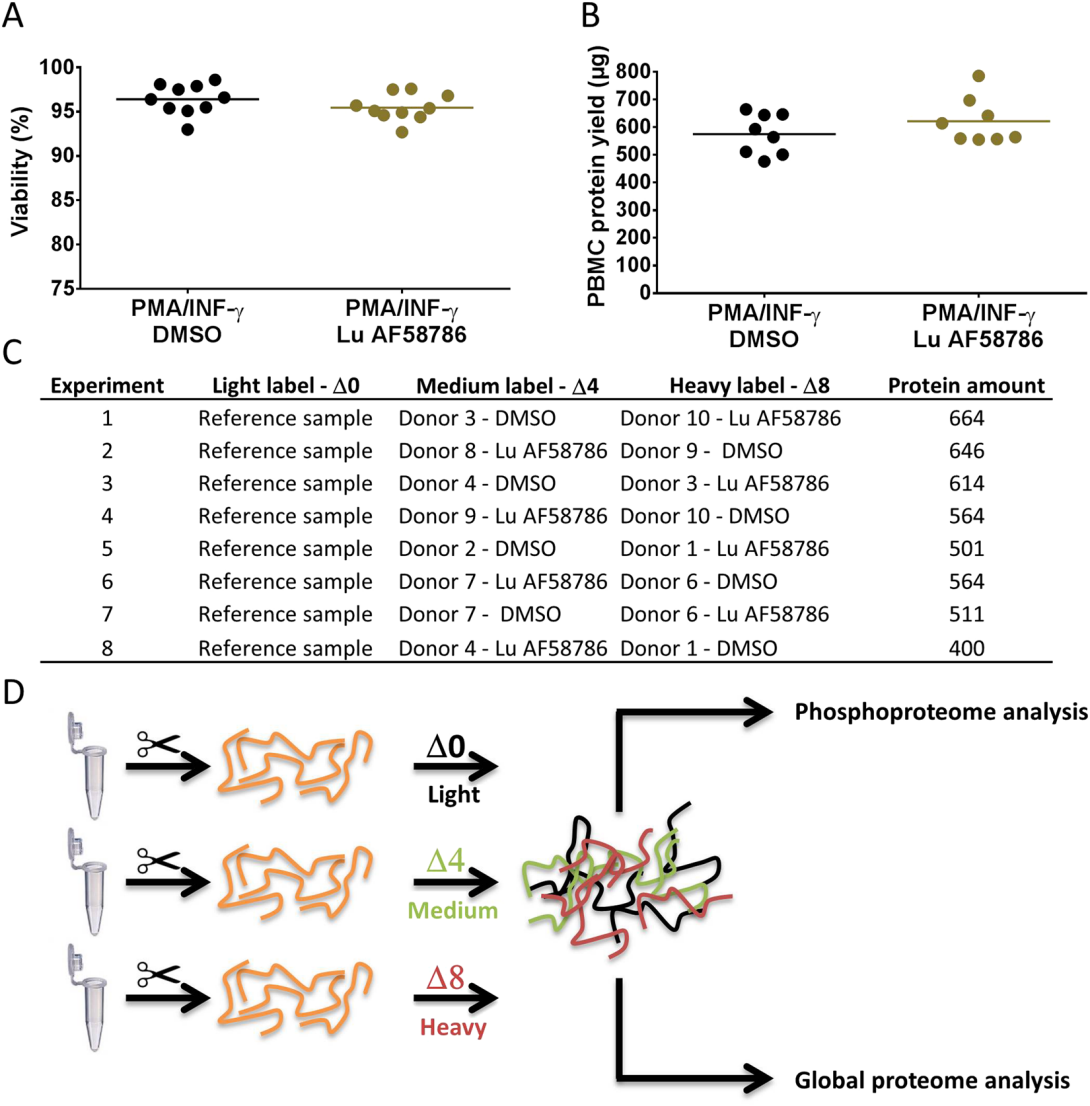
Proteomic and phosphoproteomic study to identify LRRK2 kinase activity-dependent substrates in immune-stimulated human PBMCs. (**A**) % viability after 3 DIV of human immune-stimulated PBMCs treated for 24 hrs with or without 100 nM Lu AF58786 (n = 10 donors; 2 conditions). (**B**) PBMC total protein yield (µg) after 3 DIV of human immune-stimulated PBMCs treated for 24 hrs with or without 100 nM Lu AF58786 (n = 10 donors; 2 conditions). (**C**) Experimental set-up with information on mTRAQ labelling and pairing of samples as well as amount of protein used. (**D**) Experimental flow chart of the proteomic studies. Data was analyzed by either one-way ANOVA with Dunnett’s multiple comparisons test or unpaired t-test. Data is presented as means ± SEM. PMA, phorbol 12-myristate 13-acetate; INF-γ, interferon-γ; DMSO, dimethyl sulfoxide.

Subsequent to confirming the feasibility of inducing and inhibiting LRRK2 in cultured human PBMCs large scale proteomic and phosphoproteomic studies were initiated to identify LRRK2 kinase activity dependent substrates. Human PBMCs were isolated from whole blood collected from healthy donors (n = 10 donors) and PBMC yield and viability was evaluated. As for the optimization studies there were no significant differences in PBMC yield or viability between subjects. The yield was ranging between 0.9–1.3 × 10^6^ cells/ml and viability was above 97% for all samples. For the proteomic and phosphoproteomic studies human PBMCs were isolated, cultured and immune stimulated for 3 days with or without compound treatment for 24 hrs using 100 nM of the potent and selective LRRK2 inhibitor Lu AF58786 (n = 16 samples; 10 donors). After a total of 72 hrs in culture the PBMC’s were harvested, pelleted and fresh-frozen on dry-ice. An aliquot of PBMCs was used to assess PBMC yield and viability after culturing. The average viability was above 95% and the average yield after culturing was ~6 × 10^6^ cells pr. sample (Fig. 2A,B). There were no significant differences in PBMC yield or viability between subjects and treatments. In total, PBMC cell pellets from eight DMSO-treated and eight Lu AF58786-treated samples (n = 16 samples; 10 donors) were prepared for the proteomics studies (Fig. 2C). A common reference sample was included in the proteomic studies to allow for comparison between individual experiments. Cell lysis and protein quantification showed that sufficient protein was extracted to allow for phosphoproteome and proteome analyses.

To enable quantitative mass spectrometry of human PBMCs chemical labeling with mTRAQ reagents (ABSciex) was applied subsequent to PBMC pellet extraction. Upon cell lysis of the individual cell preparations and tryptic protein cleavage peptide mixtures were desalted and peptides were then differentially isotope-labeled with mTRAQ Δ0 (light), mTRAQ Δ4 (medium) or mTRAQ Δ8 (heavy) reagents. For all samples mTRAQ labeling efficiencies were determined and found to be higher than 95% throughout the study (Supplementary Table 1), allowing the use of advanced normalization features implemented in the MaxQuant software. Subsequently, all samples were subjected to sample processing for phosphoproteomic and global proteome analysis. Equal amounts of mTRAQ labeled peptides of the three samples from a biological replicate experiment were mixed, lyophilized and then desalted. The complex mixture of tryptic peptides was thereafter fractionated by high pH reversed phase chromatography into a total of 15 fractions, which were then desalted. Aliquots of approximately 5 μg protein were taken from each fraction upon peptide desalting and were subsequently employed for proteome analysis on a LTQ Orbitrap Velos mass spectrometer. The large remainder of each fraction was enriched for phosphopeptides by means of immobilized metal affinity chromatography (IMAC), and phosphopeptide-enriched samples were then analyzed on a Q Exactive mass spectrometer. The experimental workflow for the phosphoproteomic and accompanying global proteome analysis is shown in Fig. 2D.

**Figure 3:**
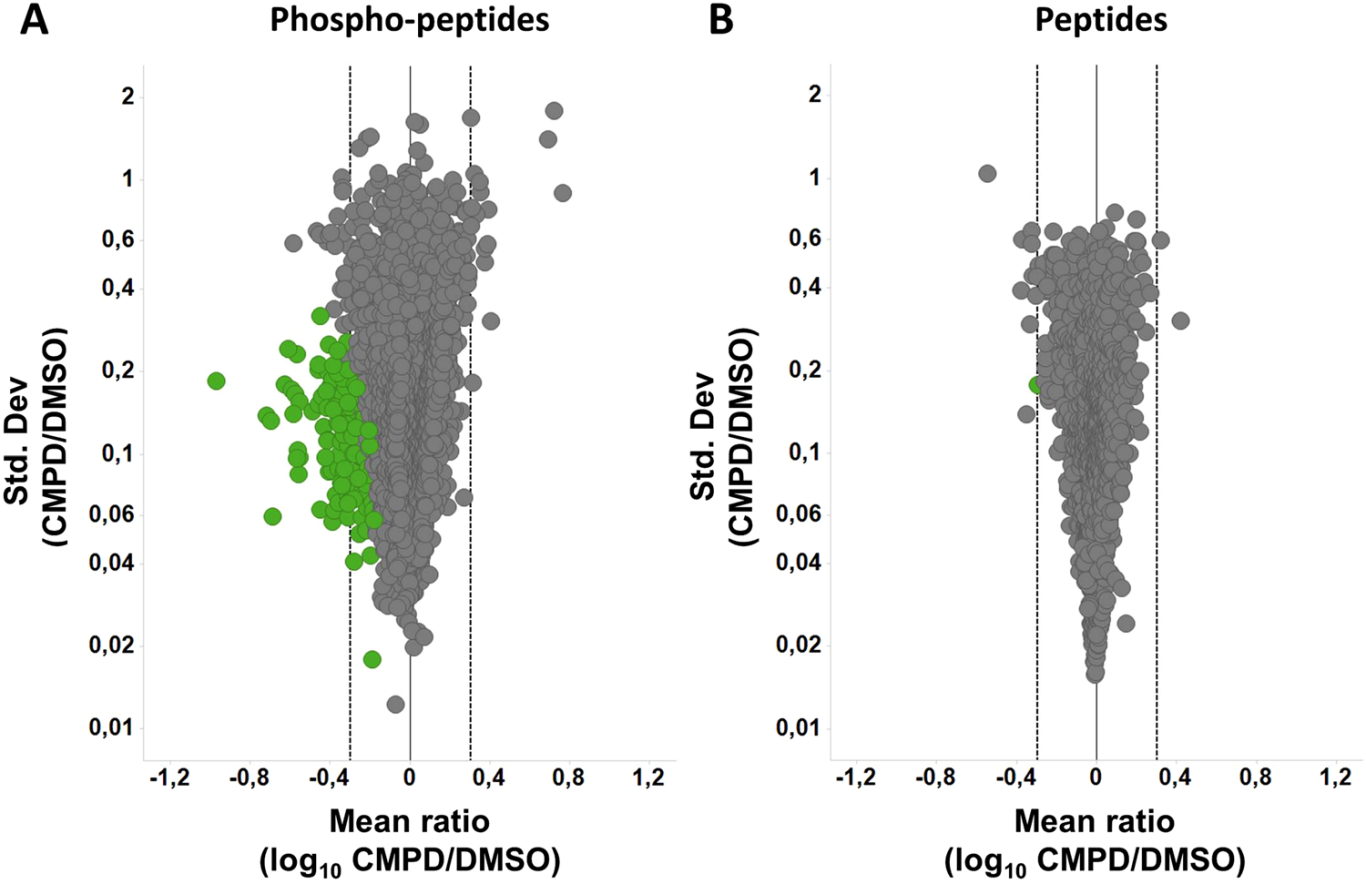
LRRK2 kinase-activity dependent phospho-petide and peptide hits identified in the phosphoproteomic and global proteomic studies. Volcano plot visualizations of the mean of log_10_-transformed CMPD/DMSO treatment ratios versus the CMPD/DMSO ratio standard deviations for (**A**) all class I phosphorylation sites or (**B**) all proteins that were quantified in at least five out of eight replicate experiments. Phosphorylation sites and proteins that are classified by the applied mean rank test to be significantly up- or down regulated are depicted in green. The black vertical line (quantified mTRAQ ratio of 1:1, 0 on log10 scale) indicates the boundary between positive (up-regulations) and negative mTRAQ ratios (down-regulations). The dashed lines indicate a two-fold regulation (±0.3 on log10 scale). CMPD, compound (Lu AF58786). DMSO, dimethyl sulfoxide.

In total, phosphoproteomic and global proteome analyses quantified more than 16,000 confidently localized phosphorylation sites and on average 7,500 proteins, indicating high phosphoproteome and proteome coverage (Table 1). Close to 11,000 phosphorylation sites and 6,000 proteins were quantified in at least five out of eight replicate experiments and were submitted to statistical significance analysis (Supplementary Files). With an accepted FDR of 5% a total of 145 class I phosphorylation sites were significantly affected by the compound treatment (Fig. 3A and Supplementary File 2). Out of these, 77 phosphorylation sites showed more than 2-fold regulation. Notably, even though no technical replicate but eight biological replicate experiments comprising a total of 16 distinct donors were conducted, we could identify significant downregulation of phosphorylation sites that changed just 1.5-fold (Fig. 3A and Supplementary File 2). This demonstrated consistent quantitative data allowing for stringent statistics across all replicate experiments. In contrast, only 1 protein, regulator of telomere elongation helicase 1 (RTEL1) was significantly affected by the compound treatment (Fig. 3B and Supplementary File 2). Interestingly, on the protein level LRRK2 was down-regulated approximately 1.7-fold (Supplementary Table 4 and Supplementary File 2). However, this effect was not statistically significant (q-value: 0.08) as there is a relatively high level of variation across the biological replicate experiments. Such reduction in LRRK2 protein amounts after LRRK2 inhibition was also observed in the optimization studies (Fig. 1G) and has previously been reported in the literature^35, 57, 58^.

**Table 1:** Number of quantified proteins and class I phosphorylation sites.

Replicate Experiment No.	Quantified Proteins (CMPD/DMSO)	Quantified proteins in min. 5/8 exp.	Quant. class-I sites (CMPD/DMSO)	Quantified class-I sites in min. 5/8 exp.
1	5.932	5.526	11.753	9.087
2	6.248	5.746	9.573	8.096
3	6.223	5.755	12.217	9.116
4	6.181	5.736	11.873	9.028
5	6.252	5.767	12.334	9.068
6	5.641	5.277	9.928	7.962
7	5.985	5.604	11.453	8.860
8	6.007	5.656	11.481	8.882
total	7.467	5.949	16.506	10.889

Enrichment analyses did not identify any enriched gene ontology terms or KEGG pathways for the set of regulated phosphoproteins (Supplementary File 2). However, network analyses revealed that the LRRK2 inhibitor treatment affected proteins which function in protein translation, the regulation of transcription and RNA processing, small GTPase activation and vesicular trafficking. Of notice, LRRK2 has been associated with all these processes^16, 41, 59–71^.

### LRRK2 kinase activity dependent phosphorylation of LRRK2, Rab10 and Rab12

**Figure 4:**
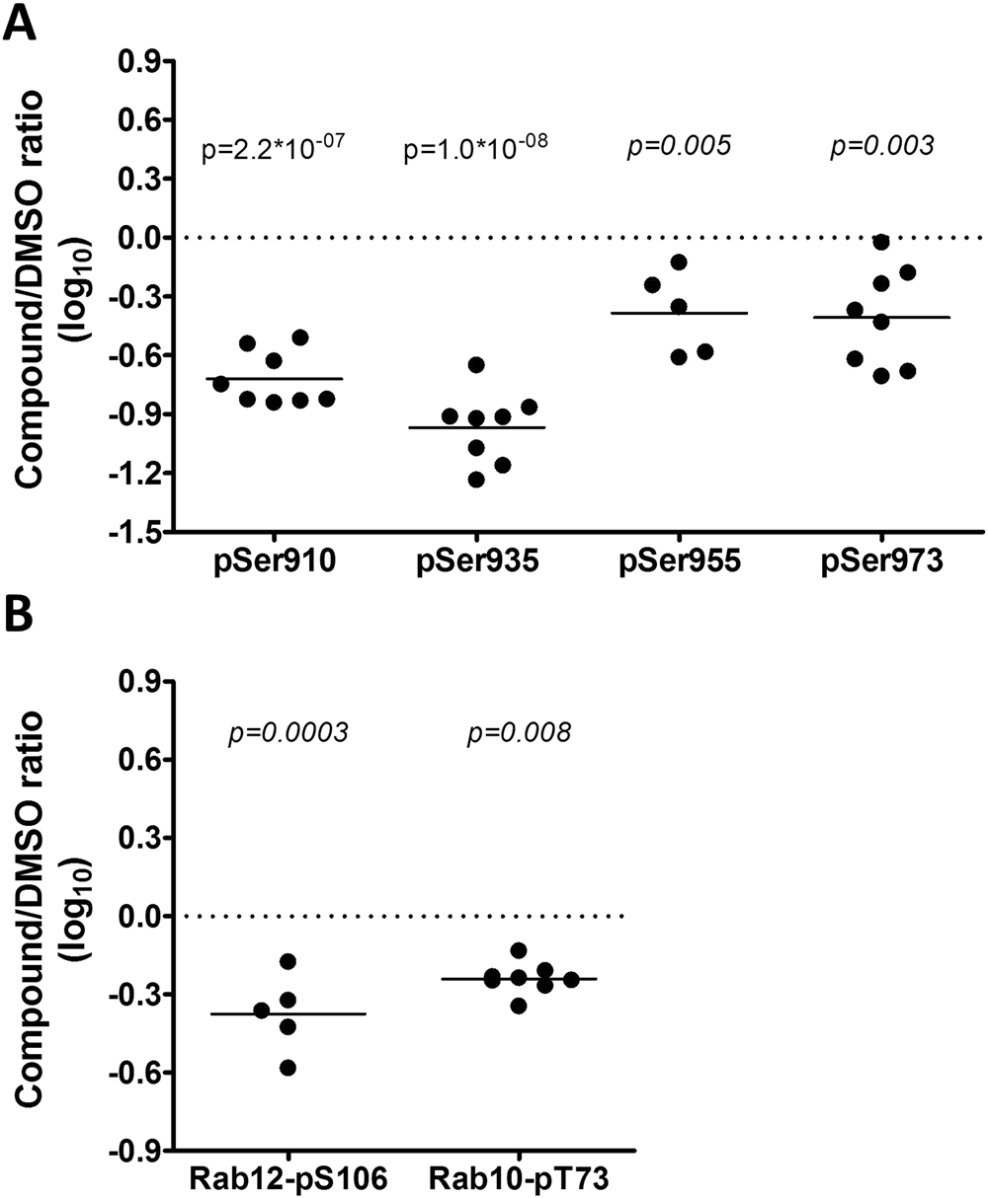
Lu AF58786 inhibits phosphorylation of LRRK2, Rab10 and Rab12 in cultured and immune stimulated human PBMCs. (**A**) LRRK2 (pSer910, pSer935, pSer955 and pSer973) phospho-site hits plotted with mean and individual replicate values of the quantified log_10_-transformed CMPD/DMSO ratio as well as the associated q-value of the performed mean rank (n = 5–8). (**B**) Rab10 (pThr73) and Rab12 (pSer106) switch II domain phospho-site hits plotted with mean and individual replicate values of the quantified log_10_-transformed CMPD/DMSO ratio as well as the associated q-value of the performed mean rank (n = 5–8). CMPD, compound (Lu AF58786). DMSO, dimethyl sulfoxide.

Several LRRK2 phosphorylation sites were among the regulated phosphoproteins. More than two-fold reductions were observed for LRRK2-pSer910, -pSer935, -pSer955 and -pSer973 sites. (Fig. 4A and Supplementary Table 4). Notably, pSer910 and pSer935 showed the largest extent of regulation throughout the dataset (Supplementary File 2). Importantly, and in support of our approach phosphorylation of both sites is known to be linked to effects of LRRK2 inhibition in heterologous expression systems and rodent models^30^)^32, 72^.

Recently, multiple Rab family GTPases have been identified as LRRK2 substrates by elaborated global phosphoproteomic analyses and were subsequently validated by *in vitro* kinase assays^41^. LRRK2 mediated phosphorylation of these Rab family proteins occurs in the conserved switch II region of the protein which is involved in GTP hydrolysis and binding to regulatory proteins^73^. Notably, our phosphoproteomics dataset comprises more than 40 distinct class I phosphorylation sites on 25 different Rab GTPase proteins that were quantified in at least 5 biological replicate experiments. This data set also comprises information for the conserved regulatory serine or threonine residue in the switch II domain of 10 distinct Rab proteins. Our statistical analyses revealed that Thr73 on Rab10 and Ser106 on Rab12 show an approximately 2-fold reduced level of phosphorylation upon treatment with the LRRK2 inhibitor while all other Rab GTPase proteins such as Rab1B, Rab7A and Rab8A were not affected in their level of phosphorylation (Fig. 4B and Supplementary Table 5). The difference in Rab10-Thr73 and Rab12-Ser106 phosphorylation between DMSO and Lu AF58786 treated PBMCs are statistically significant. Importantly, the protein amount of all Rab proteins remained unaffected by inhibitor treatment. For Rab12 and Rab10 there was no statistically significant difference in protein ratios between DMSO and CMPD-treated PBMCs suggesting that the detected down-regulation of Rab10-pThr73 and Rab12-pSer106 occurred exclusively on the phosphorylation level (Supplementary Table 5).

### Validation of phospho-specific Rab10 and Rab12 polyclonal antibodies

**Figure 5:**
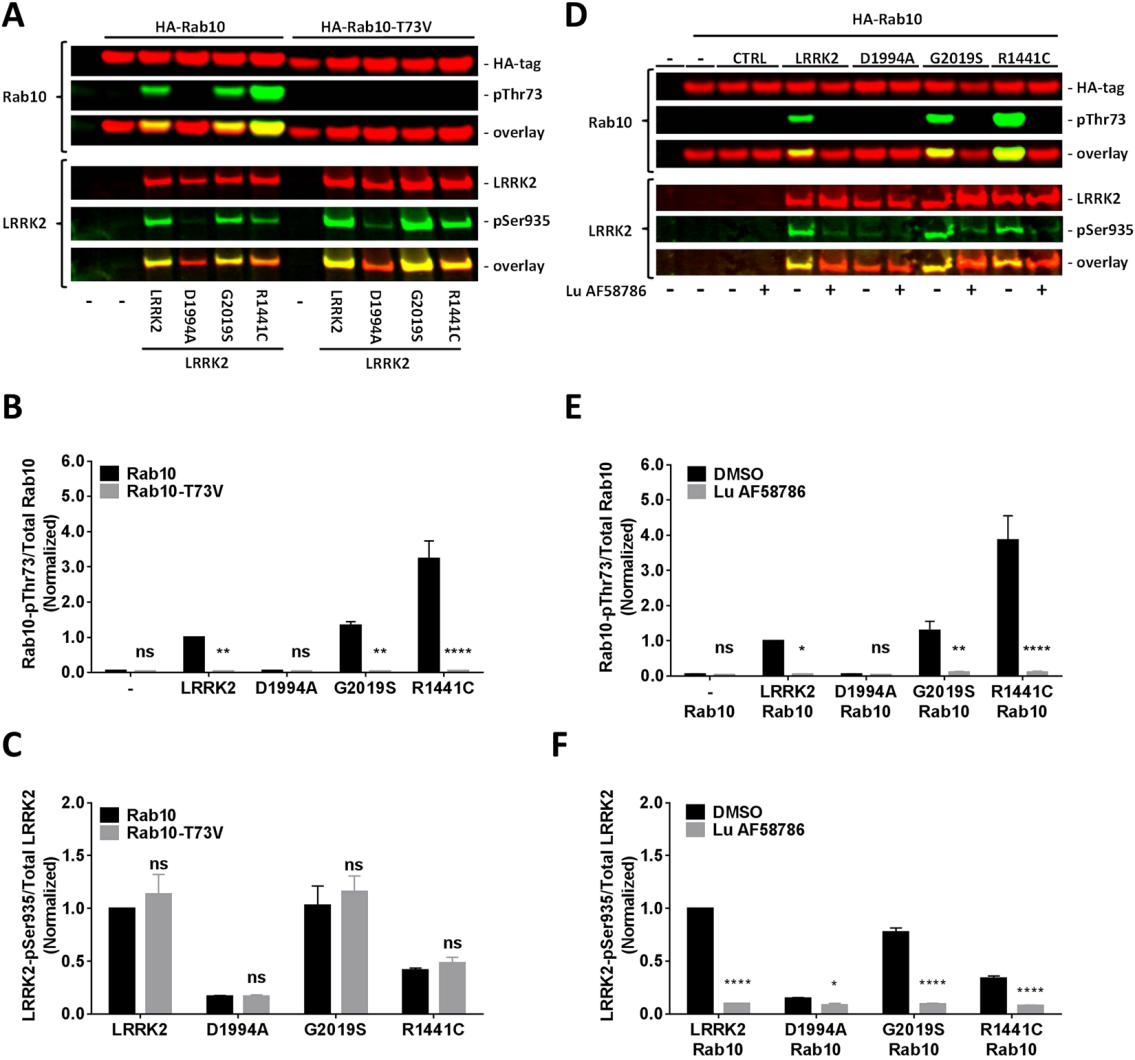
Exogenous expressed LRRK2 phosphorylates Rab10 at Thr73 in a kinase activity-dependent way in HEK293 cells. (**A**) Odyssey CLx scan Western Blot image example showing overexpressed total LRRK2 and HA-tagged Rab10 immunoreactivity (red panels), LRRK2-pSer935 and Rab10-pThr73 immunoreactivity (green panels) as well as overlay in cells overexpressing various LRRK2 exonic variants with either HA-tagged wild type Rab10 or HA-tagged phosphodeficient Rab10-T73V. *Top panel*, Rab10 and Rab10-pThr73. *Bottom panel*, LRRK2 and LRRK2-pSer935. Full-length blots are presented in Supplementary Figure 2. (**B**) Quantification of normalized Rab10-pThr73/total Rab10 ratio (n = 3 experiments). (**C**) Quantification of normalized LRRK2-pSer935/total LRRK2 ratio (n = 3 experiments). (**D**) Odyssey CLx scan Western Blot image example showing overexpressed total LRRK2 and HA-tagged Rab10 immunoreactivity (red panels), LRRK2-pSer935 and Rab10-pThr73 immunoreactivity (green panels) as well as overlay in cells overexpressing various LRRK2 exonic variants with HA-tagged wild type Rab10 and treated with either the LRRK2 inhibitor Lu AF58786 or DMSO. *Top panel*, Rab10 and Rab10-pThr73. *Bottom panel*, LRRK2 and LRRK2-pSer935. Full-length blots are presented in Supplementary Figure 2. (**E**) Quantification of normalized Rab10-pThr73/total Rab10 ratio (n = 3 experiments). (**F**) Quantification of normalized LRRK2-pSer935/total LRRK2 ratio (n = 3 experiments). Data was analyzed by two-way ANOVA with Holm-Sidak’s multiple comparisons test. Data presented as means ± SEM; *p < 0.05; **p < 0.01; ***p < 0.001; ****p < 0.0001 vs. controls (Rab10 or DMSO).

Since there are no commercially available phospho-specific antibodies targeting Rab10 and Rab12 at the indicated amino acid positions an internal production of affinity-purified rabbit polyclonal anti-Rab10-pThr73 and anti-Rab12-pSer106 antibodies was initiated (see Material and Methods). Titer determination of the affinity-purified antibodies using phospho and non-phospho peptides of Rab10 and Rab12 found that both antibodies were recognizing the phosphorylated peptides of Rab10 and Rab12 (Supplementary Tables 2 and 3). To further validate the affinity-purified antibodies crude lysates from HEK293 cells co-expressing HA-tagged human Rab10 with different human LRRK2 exonic variants were subjected to SDS-PAGE and subsequent Western Blot analyses. In cells a statistically significant Rab10 phosphorylation was detected when HA-Rab10 was co-expressed with LRRK2 WT, the kinase overactive G2019S and the causal disease variant R1441C when compared with the LRRK2 kinase dead variant D1994A suggesting that Rab10 phosphorylation in HEK293 cells is dependent on exogenous LRRK2 kinase activity (Fig. 5A–C). No Rab10 phosphorylation was observed in cells when co-expressing human HA-tagged Rab10 mutated to a valine at threonine 73 (HA-Rab10-T73V) with the various LRRK2 exonic variants suggesting that the antibody recognizes Rab10 phosphorylated at Thr73. Next, it was investigated if LRRK2 kinase inhibition impacts LRRK2-dependent Rab10 phosphorylation. Western Blot analysis detected no phosphorylation of Rab10 in crude lysates from HEK293 cells co-overexpressing HA-tagged Rab 10 and either LRRK2 WT, G2019S or R1441C that was incubated with 100 nM of the selective Lundbeck-Vernalis LRRK2 inhibitor Lu AF58786 (Fig. 5D–F). This confirms that phosphorylation of Rab10 at Thr73 in HEK293 cells is dependent on LRRK2 kinase activity. These studies parallels recent published observations using the Phos-Tag^TM^ technology to detect phosphorylated Rab10 on immunoprecipitated crude lysates from HEK293 cells co-expressing LRRK2 and HA-tagged Rab10^59^.

**Figure 6:**
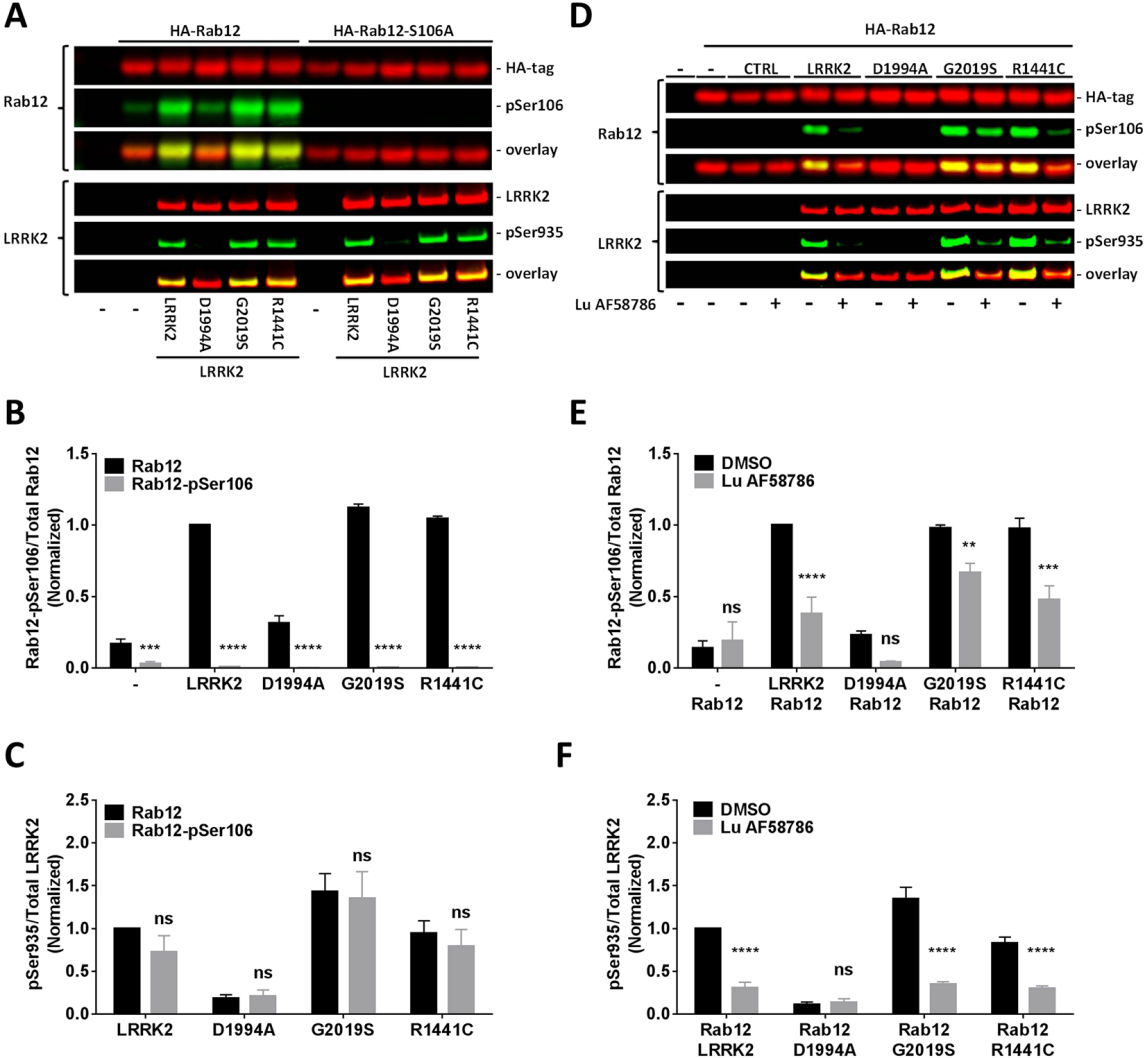
In HEK293 cells exogenous LRRK2 phosphorylates Rab12 at Ser106 in a kinase activity-dependent way. (**A**) Odyssey CLx scan Western Blot image showing overexpressed total LRRK2 and HA-tagged Rab10 immunoreactivity (red panels), LRRK2-pSer935 and Rab12-pSer106 immunoreactivity (green panels) as well as overlay in cells overexpressing various LRRK2 exonic variants with either HA-tagged wild type Rab12 or HA-tagged phosphodeficient Rab12-S106A. *Top panel*, Rab12 and Rab12-pSer106. *Bottom panel*, LRRK2 and LRRK2-pSer935. Full-length blots are presented in Supplementary Figure 3. (**B**) Quantification of normalized Rab12-pSer106/total Rab12 ratio (n = 3 experiments). (**C**) Quantification of normalized LRRK2-pSer935/total LRRK2 ratio (n = 3 experiments). (**D**) Odyssey CLx scan Western Blot image example showing overexpressed total LRRK2 and HA-tagged Rab12 immunoreactivity (red panels), LRRK2-pSer935 and Rab12-pSer106 immunoreactivity (green panels) as well as overlay in cells overexpressing various LRRK2 exonic variants with HA-tagged wild type Rab12 and treated with either the LRRK2 inhibitor Lu AF58786 or DMSO. *Top panel*, Rab12 and Rab12-pSer106. *Bottom panel*, LRRK2 and LRRK2-pSer935. Full-length blots are presented in Supplementary Figure 3. (**E**) Quantification of normalized Rab12-pSer106/total Rab12 ratio (n = 3 experiments). (**F**) Quantification of normalized LRRK2-pSer935/total LRRK2 ratio (n = 3 experiments). Data was analyzed by two-way ANOVA with Holm-Sidak’s multiple comparisons test. Data presented as means ± SEM; *p < 0.05; **p < 0.01; ***p < 0.001; ****p < 0.0001 vs. controls (Rab12 or DMSO).

Similarly, analogue studies with a rabbit polyclonal antibody targeting Rab12 phosphorylated at Ser106 also found that Rab12 was phosphorylated when co-expressed with LRRK2 exonic variants in HEK293 cells. Rab12 phosphorylation was increased after co-expression of LRRK2 WT, the kinase overactive G2019S and the causal disease variant R1441C; a much lesser effect was observed with the kinase dead variant D1994A (Fig. 6A–C). No Rab12 phosphorylation was observed in cell co-expressing human HA-tagged Rab12 mutated at serine 106 (HA-Rab12-S106A) and the various LRRK2 exonic variants suggesting that the antibody recognizes Rab12 phosphorylated at Ser106. Western Blot analysis detected a statistically significant reduction in phosphorylation of Rab12 in crude lysates from HEK293 cells co-overexpressing HA-tagged Rab 12 and either LRRK2 WT, G2019S or R1441C that was incubated with 100 nM of the selective LRRK2 inhibitor Lu AF58786 (Fig. 6D–F). The effect on Rab12 phosphorylation was not as pronounced as observed for Rab10. Thus, the results suggest that Rab12 phosphorylation on Ser106 is either partially dependent on LRRK2 kinase activity or alternatively, that the antibody might not be fully selective versus the phospho-epitope.

### Detection and inhibition of Rab10 and Rab12 phosphorylation in cultured human PBMCs

**Figure 7:**
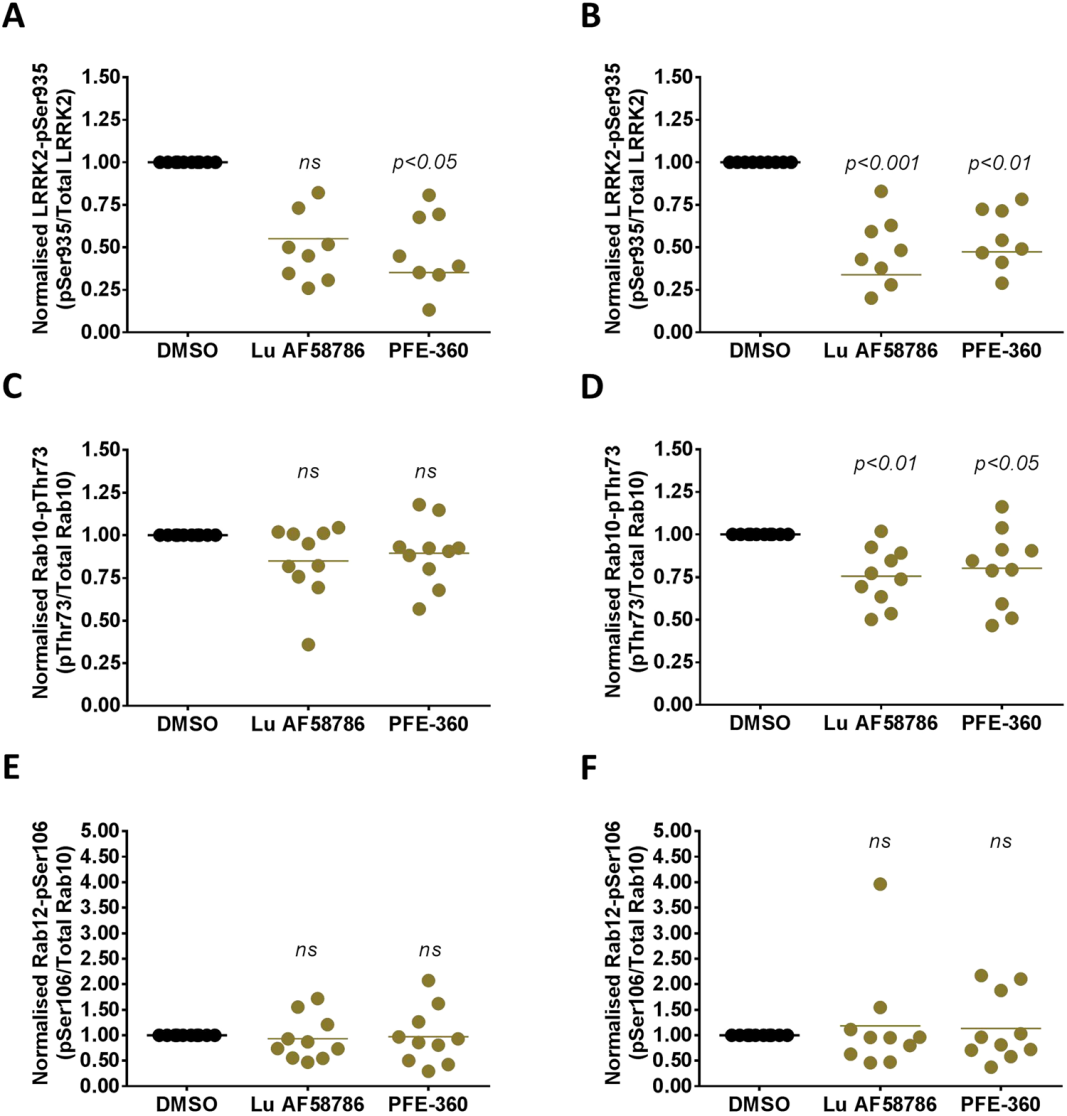
LRRK2 kinase-activity dependent LRRK2-Ser935, Rab10-Thr73 and Rab12-Ser106 phosphorylation in cultured and immune stimulated human PBMCs. Quantification of (**A**,**B**) normalized LRRK2-pSer935/total LRRK2 (**C**,**D**) Rab10-pThr73/total Rab10 and (**E**,**F**) Rab12-pSer106/total Rab10 ratios in cultured and immune stimulated human PBMCs treated with either DMSO, 100 nM Lu AF58786 or 100 nM PFE-360 (n = 10 donors; each donor in 3 conditions at *left panel*, 1hr and *right panel*, 24hrs). Data was analyzed by one-way ANOVA with Holm-Sidak’s multiple comparisons test. Data is presented as DMSO-normalized means ± SEM; p-values presented are vs. DMSO.

The studies performed in HEK293 suggested that even though LRRK2 is endogenously expressed in HEK293 cells Rab10 and Rab12 phosphorylation levels by the endogenous expressed LRRK2 are still below detection limit; whereas, increasing LRRK2 levels by overexpression led to significant increases in detectable levels of Rab10 and Rab12 phosphorylation. Likewise, Rab10 and Rab12 phosphorylation might only be detectable in stimulated human PBMCs due to increases in LRRK2 expression after PMA/INF-γ stimulation. In order to test this PBMCs from 10 different human donors were cultured 3 DIV in the presence and absence of PMA/INF-γ and the two structurally different LRRK2 inhibitor compounds Lu AF58786 and PFE-360^74^. Subsequently, cell lysates were subjected to SDS-PAGE and Western Blot analyses using antibodies targeting total LRRK2, LRRK2-pSer935, Rab10-pThr73, Rab12-pSer106 and total Rab10. LRRK2 kinase inhibition were confirmed with both inhibitors at both 1hr and 24hrs (Fig. 7A,B); although, variability between human donors was much higher than observed for the initial proteomics studies. Also, Rab10 were robustly detected in all samples; however, as observed for LRRK2-Ser935 phosphorylation some variation in the detection of phosphorylated Rab10 between samples and donors was experienced. Nevertheless, both LRRK2 kinase inhibitors were able to inhibit Rab10 phosphorylation at 24 hrs but not at 1 hr (Fig. 7C,D). Using a similar approach phosphorylated Rab12 levels were determined. Since no good antibody recognizing total Rab12 exists Rab10 levels were used for normalization. In the current study LRRK2 kinase inhibitors were not able to significant inhibit Rab12 phosphorylation at 1hr or 24 hrs (Fig. 7E,F).

### Acute inhibition of Rab10 and Rab12 phosphorylation in human PBMCs

**Figure 8:**
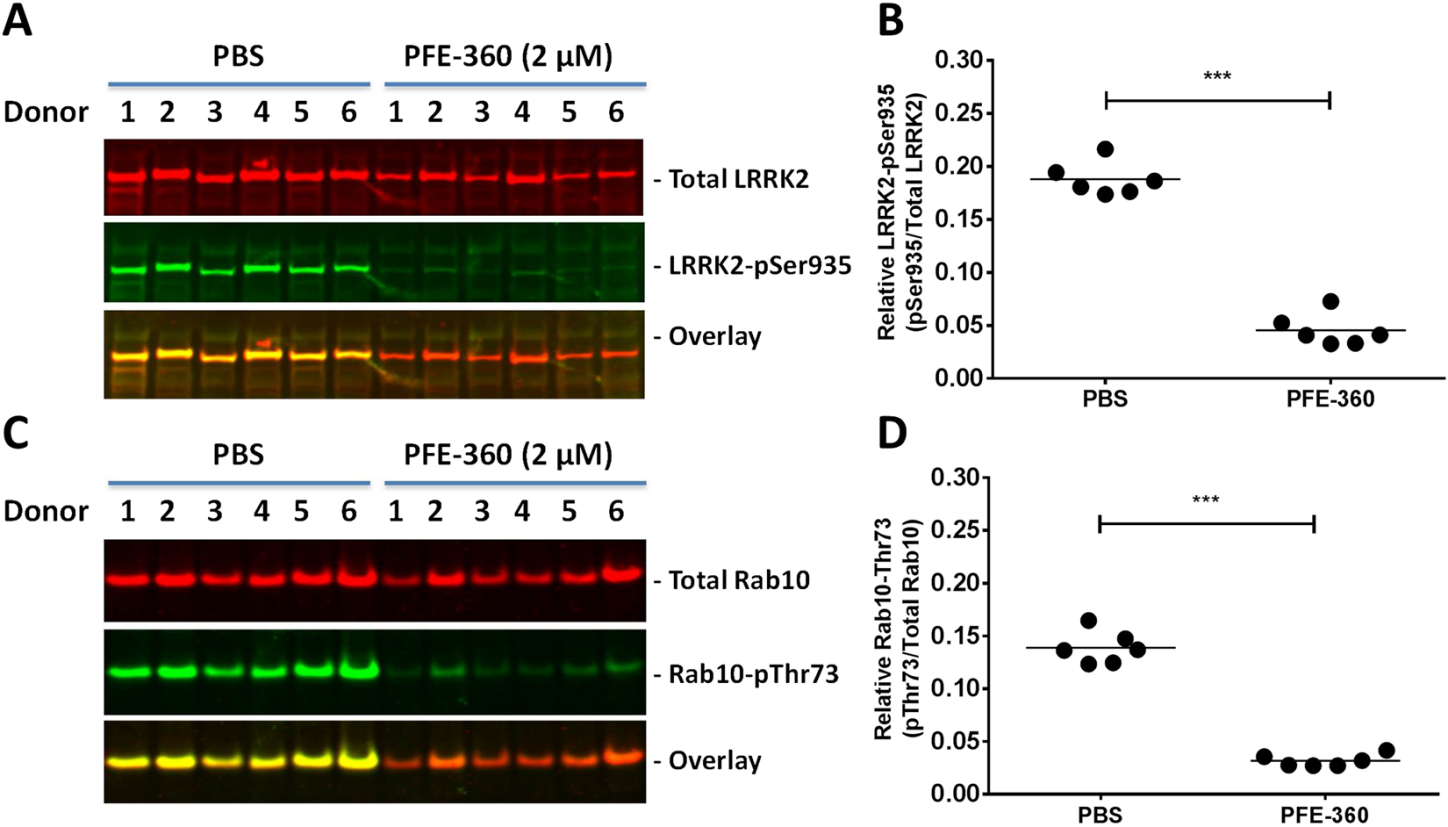
Acute LRRK2 inhibition with PFE-360 reduces LRRK2-pSer935 and Rab10-Thr73 phosphorylation in non-stimulated PBMCs from human healthy subjects. Odyssey CLx scan Western Blot images showing (**A**) LRRK2 and (**C**) Rab10 immunoreactivity (red panels), LRRK2-pSer935 and Rab10-pThr73 immunoreactivity (green panels) as well as overlay in non-stimulated human PBMCs treated with 2 µM PFE-360. Full-length blots are presented in Supplementary Figure 4. Quantification of (**B**) relative LRRK2-pSer935/total LRRK2 ratio and (**D**) relative Rab10-pThr73/total Rab10 ratio (n = 6 donors; 2 conditions). Data was analyzed by paired t-test. Data is presented as means ± SEM; ****p < 0.0001 vs. PBS.

The variability in LRRK2-pSer935, Rab12-pSer106 and Rab10-pThr73 inhibition observed in the PBMC follow-up studies were higher than observed in the initial studies. Also, *in vitro* culturing and immune stimulation of human PBMCs are not compatible with a clinical practice where human blood samples should be rapidly processed and LRRK2 inhibition subsequently measured. Thus, alternative PBMC isolation protocols that allowed for PBMC isolation and LRRK2 inhibition within a 4–8 hr time period were implemented and evaluated. In short, PBMCs were isolated from donor blood from six additional human healthy individuals. Subsequent to isolation the PBMC samples were treated for 1hr with either PBS or 2 µM of the potent and selective LRRK2 inhibitor compound PFE-360. Encouragingly, under these experimental conditions full LRRK2 inhibition was observed after 1hr of treatment. In PBMC samples treated with 2 µM PFE-360 both LRRK2-pSer935 levels (Fig. 8A,B) and Rab10-pThr73 levels (Fig. 8C,D) were significantly lower than in PBS-treated PBMC samples. To substantiate that the effect is mediated by LRRK2 inhibition the experiments were replicated with Compound A (Cmpd A) which is a selective and structurally distinct LRRK2 inhibitor compound (Supplementary Figure 1). Similar to the PFE-360 data it was found that in human PBMCs treated with 2 µM of Cmpd A LRRK2-pSer935 and Rab10-pThr73 levels were significantly lower than in PBS-treated PBMC samples (Supplementary Figure 1C,D).

**Figure 9:**
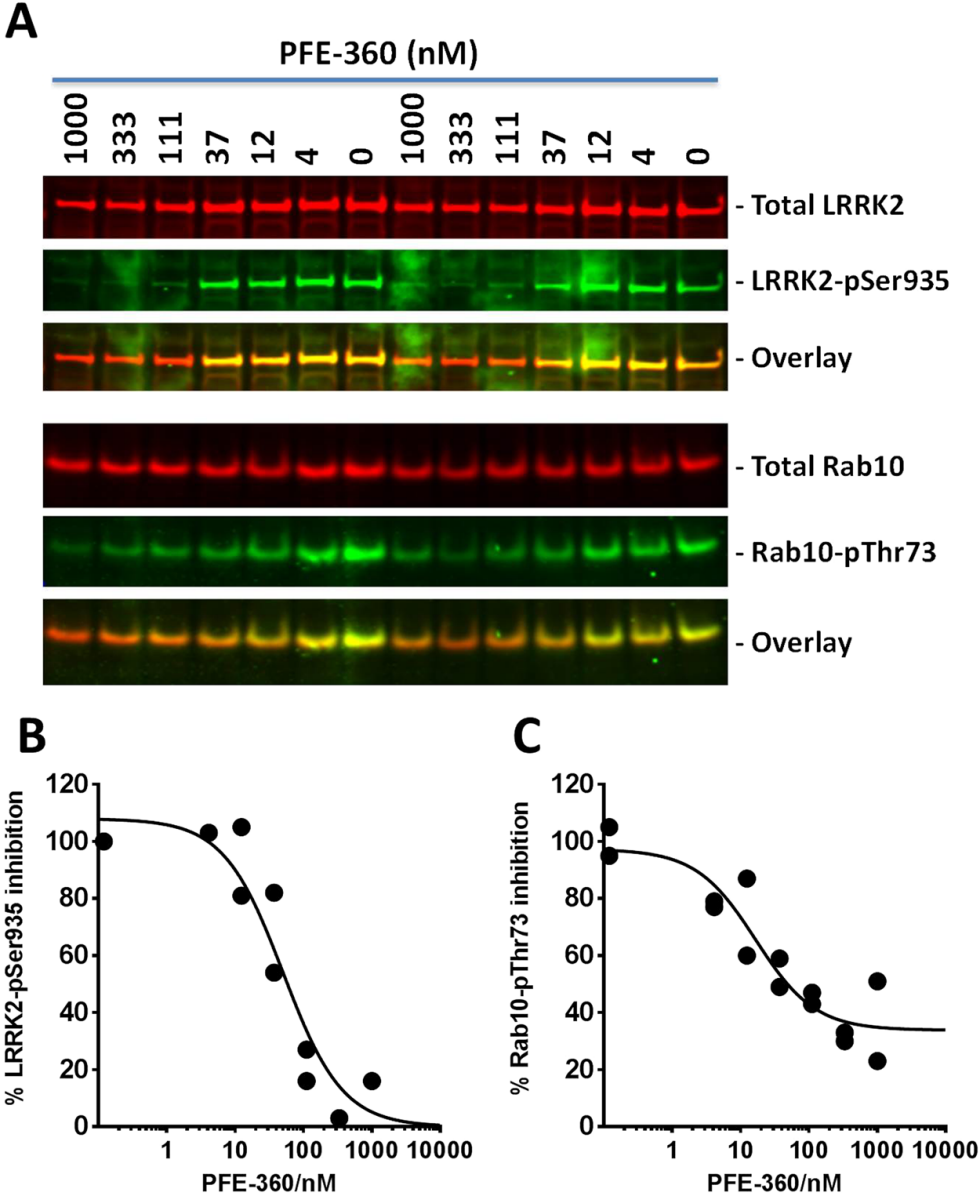
PFE-360 inhibits LRRK2-Ser935 and Rab10-Thr73 phosphorylation in a concentration-dependent manner in non-stimulated PBMCs from human healthy subjects. Determination of LRRK2 inhibitor IC_50_ values based on either Rab10-pThr73 or LRRK2-pSer935 levels in human non-stimulated PBMCs. (**A**) Odyssey CLx scan image showing Western Blot analysis of crude lysates from a pool of PBMCs from two donors treated for 1 hour with concentrations of PFE-360 ranging from 4nM-1µM. in duplicate. LRRK2 and Rab10 immunoreactivity (*red panels*), LRRK2-pSer935 and Rab10-pThr73 immunoreactivity (*green panels*) as well as overlay in non-stimulated human PBMCs. Full-length blots are presented in Supplementary Figure 5. Non-linear regression plot of percentage (**B**) LRRK2-pSer935 inhibition and (**C**) Rab10-pThr73 as a function of log10-transformed PFE-360 concentration. The experiment was repeated three times and the resulting IC50 determination is summarized in Table 2.

In follow-up PBMC studies using the same isolation and treatment protocols in three individual experiments a clear concentration-dependent effect of both PFE-360 and Cmpd A were observed on both LRRK2-pSer935 and Rab10-pThr73 inhibition (Fig. 9A–C and Supplementary Figure 2A–C). The IC_50_ for PFE-360 on LRRK2-pSer935 and Rab10-pThr73 inhibition were 24 nM and 12 nM, respectively (Table 2). No statistically significant difference between LRRK2-pSer935 and Rab10-pThr73 inhibition was observed (paired t-test; n = 3, p = 0.47). The IC_50_ for Cmpd A on LRRK2-pSer935 and Rab10-pThr73 inhibition were 266 nM and 104 nM, respectively (Table 2). No statistically significant difference between LRRK2-pSer935 and Rab10-pThr73 inhibition was observed (paired t-test; n = 3, p = 0.052).

**Table 2:** Inhibition of LRRK2-Ser935 and Rab10-Thr73 phosphorylation.

Assay	PFE-360		Cmpd A	
	IC_50_	pIC_50_ ± SEM	IC_50_	pIC_50_ ± SEM
LRRK2-pSer935	24 nM	7.6 ± 0.3 (n = 3)	266 nM	6.6 ± 0.1 (n = 3)
Rab10-pThr73	12 nM	7.9 ± 0.3 (n = 3)	104 nM	7.0 ± 0.1 (n = 3)

To rule out overlapping off-target kinase profiles of PFE-360 and Cmpd A at relevant compound concentrations kinase selectivity in acutely isolated human PBMCs were assessed by KiNativ™ profiling (Supplementary File 1). Data shows that both compounds are selective LRRK2 inhibitors with non-overlapping off-target kinase profiles at both 100 nM and 1 µM. The data also highlights that at full inhibition PFE-360 and Cmpd A are very selective LRRK2 inhibitors with no off-target kinases >60% at 100 nM and 1 µM, respectively. To further support the selectivity, KiNativ™ kinase profiling showed full inhibition of LRRK2 for PFE-360 at 100 nM and for Cmpd A at 1 µM. Both are in agreement with the Western Blot determined LRRK2-pSer935 and Rab10-pThr73 IC_50_ values for PFE-360 and Cmpd A.

## Discussion

This study is the first report of LRRK2 kinase inhibition of Rab10 and Rab12 phosphorylation in human primary cells. Previously, phosphorylation of Rab10 and Rab12 has only been observed in mouse embryonic fibroblasts (MEF) from transgenic and wild type animals, Rab10 in lung and spleen from wildtype mice and Rab12 in brain from G2019S transgenic mice as well as in heterologous expression systems such as HEK293 cells overexpressing LRRK2 variants^41, 59^. In the near future, there will be a high demand for central and peripheral pharmacodynamic markers to monitor LRRK2 kinase activity in clinical trials aiming at evaluating the potential of LRRK2 inhibitors as disease-modifying treatment for Parkinson’s disease. So far, determination of LRRK2 phosphorylation at Ser910 and Ser935 in human blood cells has been the only option. Literature suggest that phosphorylation at these sites by upstream kinases is dependent on an active ATP bound LRRK2 structure or alternatively, dephosphorylation is dependent on a non-active LRRK2 structure^30^. In support, the kinase-dead variant D1994A is phosphorylated on both sites and no significant difference has been observed between LRRK2 WT and the kinase over-active variant G2019S^68^. Thus, the pSer910 and pSer935 assays are suggested to merely represent target engagement assays rather than assays determining the degree of LRRK2 kinase activity^31, 68^. Utilizing a phosphoproteomic approach this study identified Rab10 and Rab12 as endogenous LRRK2 substrates in cultured and immune stimulated human PBMCs. Further, the identification of all four LRRK2 phosphorylation sites proximal to the LRR domain as well as identification of Rab10 and Rab12 sites indicates that the underlying phosphoproteomic study provided a solid platform for identifying and validating LRRK2 substrates in human bio-samples. In the phosphoproteomic study statistically significant phosphorylation changes as low as 1.5-fold upon LRRK2 inhibitor treatment were observed, indicating high quantification accuracy and reproducibility between the biological replicate experiments. The significance analysis revealed 145 confidently localized phosphorylation sites in total. They were all downregulated upon treatment with the LRRK2 inhibitor Lu AF58786. In addition, only one single protein, regulator of telomere elongation helicase 1 protein, was found to be 2-fold downregulated in its expression level. Using an in-house generated phospho-specific antibody targeting Rab10 phosphorylated at Thr73 it was confirmed that LRRK2 in a kinase activity dependent way phosphorylates Rab10 in a heterologous expression system as well as in cultured and immune stimulated human PBMCs. A LRRK2 kinase-activity dependent Rab12 phosphorylation using a phospho-specific antibody targeted against Rab12-pSer106 could be confirmed in a heterologous expression system but not in cultured and immune stimulated human PBMCs. This could either be due to low or highly variable levels of Rab12 expression in human PBMCs, too low levels of Rab12-Ser106 phosphorylation, low antibody-affinity towards the Rab12 phospho-epitope or combinations of the above-mentioned. This variation was also experienced when quantifying LRRK2 and Rab10 phosphorylation levels. Therefore, simplified ways of isolating PMBCs were pursued. In achieving this we were able to confirm that acute LRRK2 inhibition with the two selective and structurally distinct LRRK2 inhibitors PFE-360 and Cmpd A reduce Rab10-Thr73 phosphorylation in non-stimulated human PBMCs with apparent IC_50_’s equivalent to the LRRK2-pSer935 IC_50_’s. Importantly, this study highlights that phosphorylation of Rab10 in human primary cells can provide an alternative option to pSer910 and pSer935 target engagement assays for determining pharmacodynamic effects of LRRK2 inhibition in human PBMCs. Importantly, we also show that both the abovementioned LRRK2 inhibitor compounds have non-overlapping off-target kinases in human PBMCs at 1 µM concentrations. Since the kinase selectivity experiments were conducted at a 2-fold lower concentration than the acute LRRK2 inhibition studies we cannot completely rule out a larger overlap in kinase selectivity profiles of PFE-360 and Cmpd A in PBMCs at 2 µM. Nonetheless, for PFE-360 we observe similar Rab10-pThr73 inhibition at both 100 nM and 1 µM (Fig. 9) and for Cmpd A similar Rab10-pThr73 inhibition at 1 µM and 3 µM (Supplementary Figure 2) suggesting no involvement of other kinases than LRRK2 on Rab10-Thr73 inhibition at those compound concentrations. Together, this strongly supports that Rab10 is phosphorylated at Thr73 by LRRK2.

Our findings in cultured human PBMCs also validate recent observations obtained in animal models and heterologous expression systems by Steger *et al*. suggesting that several Rab GTPases including Rab10 and Rab12 are LRRK2 kinase substrates^41^. Rab10 and Rab12 belong to the family of small Rab GTPases. These proteins are known to be master regulators of intracellular vesicular trafficking^75^. Besides Rab10 and Rab12 also Rab8A and Rab7L1 were proposed as LRRK2 substrates by Steger *et al*.^41^. This is of particular interest since Rab7L1 is part of the LRRK2 interactome^65, 76^, Rab7L1 deficient mice show kidney abnormalities^60^ similar to those observed in LRRK2 KO mice and in GWAS single nucleotide polymorphisms in the gene encoding the small Rab GTPase *RAB7L1* have been shown to genetically interact with the *LRRK2* loci to modulate risk of Parkinson’s disease^10, 77^. Another PD-associated kinase *PINK1* is found to mediate indirect phosphorylation of Rab8A at position Ser-111 thereby regulating Rab8A activation^78^ and accumulating evidence also links the α-synuclein biology to several Rab GTPases here including Rab8A^79–87^. Thus, involvement of Rab GTPase and intracellular vesicular trafficking may prove to underlie neurodegenerative mechanisms associated with some aspects of α-synuclein pathology in PD.

Rab10 is expressed in various tissues and organs including brain, spinal cord, skeletal muscle, lung, kidney and heart^88^. It is localized to early endosomes where it regulates vesicular recycling of cargo molecules to and from the plasma membrane^89–92^. In macrophages Rab10 expression is induced upon TLR4 stimulation suggesting a functional role for Rab10 in immune stimulated mononuclear peripheral blood cells^93^. Rab10 is involved in membrane transport in kidney cells^94, 95^, has been functionally coupled to autophagic processes and interestingly, genetic ablation of *RAB10* leads to accumulation of lipid droplets in hepatocytes^96^. LRRK2 is not expressed in the liver; however, the latter observation is not unlike observations of lipid droplet accumulations in the kidneys of LRRK2 KO rats and mice suggestive of Rab10 playing a functional role in LRRK2 kinase activity dependent mechanisms^34, 35, 97^. This study shows that Rab10 phosphorylation is dependent on LRRK2 kinase activity in human PBMCs. Further, compared to LRRK2 WT the disease variants G2019S and R1441C exhibits increased pThr73 levels in HEK293 cells (Fig. 6A–E) thereby confirming literature reports that *LRRK2* exonic variation associated with Parkinson’s disease impacts the level of Rab10 phosphorylation^41^. Such observations have not yet been confirmed in human primary cells derived from carriers of LRRK2 disease variants such as G2019S. Our study provided various additional candidates as LRRK2 *in vivo* substrates and downstream targets. Further exploration of these phosphorylation sites would evaluate whether other phospho-proteins besides Rab10 and Rab12 could serve as pharmacodynamic markers for *in vivo* and *ex vivo* LRRK2 target engagement and inhibition. For now the most promising candidate is Rab10 phosphorylated at the Thr73 position in human blood cells. Further, *in vivo* studies in rodent models carrying wild type and disease-related Lrrk2 variants such as the G2019S as well as *ex vivo* studies in human PBMCs from G2019S carriers and non-carriers will confirm and validate whether Rab10-pThr73 is useful as a surrogate marker for LRRK2 kinase activity. If proven so, such a marker could be very valuable for stratifying PD patients eligible for LRRK2 inhibitor clinical trials.

## Methods

### LRRK2 inhibitor compounds

Lu AF58786 was synthesized at Vernalis (R&D) Ltd (Example 14, WO 2014170248). PFE-360 was synthesized at H. Lundbeck as described in patent application US2014/005183 (Example 217). The structure of compound A (Cmpd A) cannot be disclosed at this stage. The structure will be published in an upcoming publication.

### Ethical statement

All studies on human donor blood samples were carried out in accordance with guidelines and regulations of Danish legislation. Blood was donated at the Danish Bloodbank at Hvidovre Hospital (Copenhagen, Denmark). At the donation site, all human donor blood samples were fully anonymized and informed consent was obtained from all human subjects. According to Danish legislation, experiments performed on anonymized human bio samples do not require approval from a local ethics committee. All subsequent experiments were performed at Lundbeck (Valby, Denmark) according to guidelines and regulations of Danish legislation.

### Blood samples

Donor blood from human healthy subjects were collected at the Danish Bloodbank at Hvidovre Hospital in 9 mL K2-EDTA tubes (Vacutainer BD 367525). After blood collection, tubes were immediately turned-over several times to mix blood and anticoagulant. The samples were shipped at controlled temperature ranging from 15–25 °C. Receipt of samples was within 2 h following blood collection.

### Isolation of peripheral blood mononuclear cells (PBMCs) for culturing

PBMC were isolated from fresh blood collected within a maximal time of 4 hours after blood collection. Leucosep tubes (Greiner 227290) was added 15 ml Ficoll (GE Healthcare 17-440-03) and spun 30 s at 1000 × g. Blood was diluted 1:1 using D-PBS 1X (Gibco 14190), transfered to the Leucosep and centrifuged at 800 g for 15 min without brake. The upper layer corresponding to the diluted plasma was removed and the PBMC layer collected using a pipette or by pouring into a clean BD Falcon 50 ml tube. Cells were re-suspended and washed four times with 10 ml of D-PBS 1X (Gibco 14190) followed by centrifugation at 250 g for 10 min with brake at room temperature. The supernatant was discarded and the cells re-suspended in Complete medium equilibrated to 37 °C. The number of dead and living cells were counted on a Nucleocounter and used to calculate percent viability.

### Culturing and immune stimulation of human PBMCs

Freshly isolated PBMCs were plated at a density ranging from 7–20 × 10^6^ cells in 10 cm petri dishes (Costar 3262) in RPMI-1640 media (Gibco 61870–010) supplemented with 10% non-heat inactivated FBS, 5 ml Antimycotic (Sigma cat:5965) with or without an immune stimulation cocktail consisting of 400 nM phorbol 12-myristate 13-acetate (PMA) and 100 ng/ml interferon-γ (INF-γ). After 48 hrs in culture the cultures were added a fifth of the volume media containing 5x the final concentration of the respective LRRK2 inhibitor with or without PMA and interferon-γ. For the PBMC optimization studies the experimental conditions were as follows: (1) control – plated, un-induced and untreated (n = 10); (2) immune stimulated - induced with PMA/INFγ for 72 hrs (n = 10) and (3) immune stimulated and compound treated - induced with PMA/INF-γ for 72 hrs and treated with the potent and selective LRRK2 kinase inhibitor Lu AF58786 for the last 24 hrs (n = 10). Approximately, 400 µg of protein from each individual donor was needed in order to run the full PBMC stimulation and inhibition study with subsequent global proteome and phosphoproteome (PhosphoScout®) analyses. After 72 hrs in culture the PBMC’s were harvested, pelleted and fresh-frozen on dry-ice. An aliquot of PBMCs was used for assessment of PBMC yield and viability. For the PBMC proteomic studies the following experimental conditions were used: (1) immune stimulated - induced with PMA/INFγ for 72 hrs (7 donors; n = 7 samples); (b) immune stimulated and compound treated - induced with PMA/INF-γ for 72 hrs and treated with the LRRK2 kinase inhibitor Lu AF58786 for the last 24 hrs (7 donors; n = 7 samples).

### KiNativ profiling of human PBMCs

Compounds were shipped to ActivX Technologies (San Diego, US) where LRRK2 target engagement and broad kinase profiling was performed using KiNativ™ procedures. Live cell treatment of human peripheral blood mononuclear cells (PBMCs) at two compound concentrations (1 µM and 0.1 µM) were used to evaluate and compare *in vitro* target engagement and kinase selectivity, respectively (see Supplementary File 1).

### Proteomic and phosphoproteomic studies – sample preparation

Upon the respective treatments cells were washed with cold PBS, snap-frozen in liquid N_2_ and shipped to Evotec on dry-ice. Subsequent sample preparation for MS analysis was executed by Evotec Munich. Cell lysis was performed in ice-cold lysis buffer (8 M urea, 50 mM Tris pH 8.2, 10 mM sodium pyrophosphate, 5 mM EDTA, 5 mM EGTA, 10 mM sodium fluoride, 10 mM β-glycerophosphate, 10 mM sodium orthovanadate, phosphatase inhibitor cocktail 2 and 3 (Sigma, 1:100 (v/v)) and Complete protease inhibitor cocktail tablets (Roche). Cell extracts were sonicated on ice and the cell debris was removed by centrifugation before protein concentrations were determined (Bradford, BioRad).

Each sample was reduced with 10 mM dithiothreitol for 30 min and thereupon alkylated in the presence of 55 mM iodoacetamide for 30 min in the dark. Subsequently, endoproteinase Lys-C (Wako) was added at an enzyme-to-substrate ratio of 1:100 and incubated for 2 h at room temperature. Samples were thereafter diluted 1:4 with 20 mM Tris-HCl pH 8.2 before adding trypsin (Promega) at an enzyme-to-substrate ratio of 1:100 followed by overnight incubation. The resulting peptide mixtures were acidified by the addition of trifuoroacetic acid (TFA) to a final concentration of 0.5% and subsequently desalted using C18 Sep-Pak columns (100 mg sorbent weight, Waters). Peptides were eluted with 50% acetonitrile, 0.5% acetic acid, and aliquoted into the respective fraction that was utilized for the individual replicate experiment and/or for the pooled reference sample. Aliquoted samples were snap-frozen in liquid nitrogen and lyophilized.

All samples were then chemically labeled with the respective mTRAQ isotopic variants (ABSciex) according to the manufacturer’s instructions. The labeling efficiencies were determined for each sample and found to be above 95% for all samples (Supplementary Table 1). Equal amounts of differentially mTRAQ-labeled peptides were then combined, frozen in liquid nitrogen, lyophilized and desalted using C18 Sep-Pak columns (100 mg sorbent weight, Waters). For sample decomplexing peptides were then applied to chromatographic fractionation by high pH reversed phase based on a publication by Wang *et al*.^98^. Briefly, peptides were reconstituted in 20 mM ammonium formate (pH 10, buffer A), loaded onto an XBridge C18, 200 × 4.6 mm analytical column (Waters) operated with the Äkta Explorer system (GE Healthcare) and separated by applying a segmented gradient increasing the acetonitrile concentration from 7% to 30% buffer B (buffer A supplemented with 80% acetonitrile) over 15 min followed by a 5 min gradient to 55% over 5 min. For each individual experiment the collected peptide fractions were then combined in a non-linear way to generate 15 samples with equal peptide amounts. Fractions were frozen in liquid nitrogen, lyophilized, reconstituted in 0.1% TFA and desalted using C18 Sep-Pak columns (100 mg sorbent weight, Waters). An aliquot of each fraction corresponding to approximately 5 μg peptide starting material was removed for global proteome analysis. The large remainder of each fraction was frozen in liquid nitrogen and lyophilized and subsequently applied to phosphopeptide enrichment. Enrichment of phosphopeptides was performed according to a protocol by Steven Carr and coworkers^99^. Briefly, peptides of each fraction were reconstituted in IMAC loading buffer (80% acetonitrile containing 0.1% TFA) to reach a final concentration of approximately 5 mg/ml. To generate the IMAC-resin Ni ions of Ni-NTA Superflow Agarose Beads (Qiagen) were removed by incubation with 100 mM EDTA and subsequently replaced by Fe^3+^ ions. The resin was thereupon washed with H_2_O and reconstituted in a 1:1:1 mix of acetonitrile, methanol and 0.01% acetic acid. To each sample 10 μl of equilibrated IMAC resin was added and incubated for 30 min at 25 °C and 1,400 rpm in a Thermomixer (Eppendorf). This slurry was then loaded onto in-house build C18 StageTips columns^100^. Subsequently beads were washed with IMAC loading buffer and phosphopeptides were eluted onto the C18 material by washing twice with a 500 mM K_2_HPO_4_ solution. The C18-bound phosphopeptides were washed with 0.1% formic acid, eluted with 50% ACN, 0.1% FA, concentrated in a VacufugeTM (Eppendorf) and reconstituted in 0.1% FA before MS analysis.

### Proteomics and phosphoproteomic studies – MS analysis

All LC-MS/MS analyses in the phosphoproteomic (PhosphoScout®) experiments were performed on a Q Exactive mass spectrometer (Thermo Fisher Scientific) equipped with an Easy nLC-1000 UPLC system (Thermo Fisher Scientific). Phosphopeptide-enriched samples were loaded with an auto sampler onto a 40 cm fused silica emitter (New Objective) packed in-house with reversed phase material (Reprusil-Pur C18-AQ, 1.9 μm, Dr. Maisch GmbH) at a maximum pressure of 950 bar. The bound peptides were eluted over 125 min run time and sprayed directly into the mass spectrometer using a nanoelectrospray ion source (ProxeonBiosystems). The mass spectrometer was operated in the data dependent mode to automatically switch between MS full scans at a resolution R = 70,000 (at m/z  200) with a target value of 3,000,000 counts (max. Injection time = 45 ms) and MS/MS fragmentation scans at R = 35,000 and target value of 200,000 ions (max. Injection time = 120 ms). The ten most intense peptide ions were selected higher-energy collisional dissociation (HCD).

Mass spectrometric analyses for the accompanying global proteome analysis was performed with a LTF-Orbitrap Velos (Thermo Fisher Scientific) equipped with an Easy nLC-1000 UPLC system (Thermo Fisher Scientific). Samples were loaded as described above.The mass spectrometer was operated in the data dependent mode to automatically switch between MS full scans at a resolution R = 60,000 (at m/z 200) with a target value of 1,000,000 counts (max. Injection time = 500 ms). The fifteen most intense peptide ions were selected for collision induced fragmentation and the resulting fragmentation spectra were recorded in the linear ion trap at a target value of 5000 ion counts (max. Injection time = 25 ms). The resulting fragmentation spectra were recorded in the linear ion trap.

### Proteomics and phosphoproteomic studies – data processing

All raw files acquired in this study were collectively processed with the MaxQuant software suite (version 1.5.3.31) for peptide and protein identification and quantification using a human uniprot databases (version 09 2015)^101^. The maximum mass deviations allowed for MS and MS2 peaks were 6 ppm and 0.5 Da for data acquired with the LTQ-Velos and 4.5 ppm and 20 ppm for data acquired with the Q Exactive. Carbamidomethylation of cysteine was set as a fixed modification and oxidation of methionine, protein N-terminal ﻿acetylation were allowed as variable modifications. In addition, for phosphopeptide-enriched samples also phosphorylation of serine, threonine and tyrosine residues were allowed as variable modifications. All peptides were required to have a minimum peptide length of seven amino acids and a maximum of two missed cleavages and three labeled amino acids were allowed. The false discovery rate (FDR) for protein and peptide identifications of set to 1% and the match between runs option was enabled.

### Proteomics and phosphoproteomic studies – statistical and bioinformatics data analysis

The resulting list of phosphorylation sites exported from the MaxQuant software was filtered for class-I sites, i.e. phosphorylation sites that could be located with high confidence^102^. For the comparison of cmpd versus DMSO treated PBMC the ratios were calculated via the respective pooled reference sample (light mTRAQ label) for each individual replicate experiment and a hypothesis test was conducted to identify significantly regulated phosphorylation sites. For these analyses, only phosphorylation sites that were quantified in at least five out of the eight replicate experiments were used. Significantly regulated sites were identified with the mean rank test using an estimated FDR of 5%^103^. Similar analyses were performed with the global proteome data to identify proteins that became significantly regulated upon the different treatments.

Enrichment analyses of gene ontology (GO) terms and KEGG pathways were performed by applying Fisher’s exact test and utilizing all proteins with significantly altered class I phosphorylation sites as input.

For a network analysis all proteins with significantly regulated phosphorylation sites (FDR <5%) were mapped onto the STRING protein-protein network version 10^104^. Only high confidence interactions (confidence score >0.7) were considered. Proteins without any interactions satisfying the above criteria were removed from the network.

### Generation and validation of phospho-specific Rab10 and Rab12 antibodies

Phospho-specific Rab10 and Rab12 antibodies targeted against Thr73 and Ser106, respectively, were generated at Genscript using a customized “Phospho-specific Polyclonal Antibody Package”. For each antibody project four rabbits were immunized at least three times using either Rab10-Thr73 or Rab12-Ser106 phosphopeptides coupled to a KLH conjugate. The sequences used for immunization were ERFH(pT)ITTSYYRC and ERFN(pS)ITSAYYRSAKC for Rab10 and Rab12, respectively. Subsequent to the 3rd immunization titer determination of phosphopeptide and non-phosphopeptide affinities guided the selection of rabbits for small-scale bleed, affinity purification as well as indirect ELISA determination of antibody specificity and selectivity. Coating antigens used for the indirect ELISA were the phosphopeptides or non-phosphopeptides. The antigens were diluted in Phosphate Buffered Saline (PBS) at pH 7.4 and used in a coating concentration and volume of 4 μg/ml and 100 μl/well. The secondary antibody used for quantification was a peroxidase-conjugated goat anti-rabbit IgG. Finally, rabbits proceeded towards final boost, bleed and affinity purification of the antibodies. The ELISA results for the affinity purified Rab10-pThr73 (#5981) and the Rab12-pSer106 (#5919) antibodies are listed in Supplementary Tables 2 and 3. Both antibodies show high specificity and selectivity towards the phospho-peptide.

### Cell culture and transfections

Plasmids encoding various LRRK2 and Rab GTPase protein variants were transiently expressed in HEK293 cells using Lipofectamine®2000 according to the manufacturer’s instructions (Thermo Fisher Scientific, US).

### Lu AF58786 IC50 determination in LRRK2 WT, G2019S and A2016T expressing HEK293 cells

Cell-based IC_50_ of Lu AF58786 was determined using a cell-based quantitative immunocytochemistry 96-well ICW assay based on LRRK2-Ser935 phosphorylation using theOdyssey CLx near infra-red technology (LI-COR, Nebraska). HEK293 cells grown in clear-bottom 96-well plates were transfected with either wild type LRRK2, kinase overactive variant G2019S, inhibition-resistant mutant A2016T or mock, respectively. One hour prior to cell fixation plates were pre-incubated with various concentrations of Lu AF58786 to inhibit LRRK2-Ser935 phosphorylation. Subsequently, the plates were processed as previously described^105^.

### SDS-PAGE and Western Blotting

HEK293 cells were plated at a density of 1 × 10^6^ cells/well in 6-well plates pre-coated for 1 hr at 37 °C with poly-l-lysin. Subsequent to the transient transfection HEK293 cells were harvested using 1 mL cold Phosphate Buffered Saline (PBS) buffer (Invitrogen, California, US) and subsequently centrifuged at 800 g for 2 minutes. Cell pellets were resuspended and solubilized in 100 µL lysis buffer (50 mM Tris hydrochloride, 1 mM magnesium chloride, 1% Triton, 0.1% sodium dodecyl sulfate (SDS), pH 8.0) on ice for 20 minutes and then centrifuged at 20,000 g for 30 minutes at 4 °C. The solubilized crude cell lysate was size mobility separated by SDS-polyacrylamide gel electrophoresis (SDS-PAGE). LRRK2 was separated on a 3–8% Tris-Acetate gel (NuPAGE® Tris-Acetate Mini Gels, Life Technologies, Paisley, UK) and Rab10 and Rab12 were separated using a NuPAGE® Novex 4–12% Bis-Tris Gel (Invitrogen, California, US). An amount of 10 µg total protein was loaded in each well. Proteins were transferred to immobilon-FL PVDF membranes (Millipore, Billerica, US). Membranes were incubated with primary antibodies overnight at 4 °C: mouse monoclonal [N241A/34] anti-LRRK2 antibody (1:2,000; NeuroMab, California, US), rabbit monoclonal [UDD2 10(12)] anti-pS935-LRRK2 antibody (1:1,000; RabMAb®, Abcam, Cambridge, UK), mouse anti-HA monoclonal antibody [clone HA-7] (1:10,000; Santa Cruz Biotechnology, Texas, US), rabbit anti-Rab10-pThr73 polyclonal [#5981] (1:2,000; H. Lundbeck A/S, DK) and rabbit anti-Rab12-Ser106 polyclonal antibody [#5919] (1:5,000; H. Lundbeck A/S, DK). Subsequently, the membranes were washed and incubated with secondary antibodies for 1 hour at room temperature: Anti-rabbit IgG F(c) (GOAT) antibody IRDye® 800CW Conjugated (1:10,000; Rockland Immunochemicals Inc., Gilbertsville, US) and anti-mouse Alexa Fluor® 680 Goat anti-mouse IgM (1:20,000; Life Technologies, Paisley, UK). The proteins were visually detected by infrared imaging using Odyssey CLx (LI-COR, Nebraska, US). Membranes were scanned and band intensities were quantified using the Image Studio version 3.1.4 (LI-COR, Nebraska).

### Isolation of PBMCs for acute inhibition studies

PBMCs were isolated using the density gradient medium, Lymphoprep™ from Stemcell Technologies (Vancouver, Canada) following their standard protocol. Briefly, 4 ml blood was diluted with 4 ml PBS and applied at the SepMate™ tubes (Stemcell Technologies) and spun at 1200xg for 20 min at RT in the centrifuge without brakes. The PBMC layer was collected and spun at 600xg for 8 min and washed two times in PBS by spinning at 600xg between each wash. After the last wash, the cells were counted and the cellular concentration was adjusted to 50 × 10^6^ cells/ml in PBS. Donor samples were treated with the LRRK2 inhibitor PFE-360 for one hour and the cells were hereafter lysed by adding 1/10 volume lysis buffer (5% Triton-X, 5% Digitonin, 10 mM EDTA) containing phosphatase and protease inhibitor cocktail (Roche Diagnostics, Mannheim, Germany). The lysates from human PBMCs were subjected to SDS-PAGE and Western Blotting procedures as described above.

### Data analysis and statistics

Data and statistical analyses were performed using Prism 5 (GraphPad Software, USA) Data were analyzed by either 1-way or 2-way analysis of variance (ANOVA) or by t-test. Post-hoc tests following ANOVAs were conducted using Dunnett’s or Holm-Sidak’s multiple comparisons test. Two-tailed levels of significance were used and p < 0.05 was considered statistically significant.

## Electronic supplementary material

Supplementary Data File

Supplementary File 1

Supplementary File 2

## References

[1] Aasly JO (2005). Clinical features of LRRK2-associated Parkinson’s disease in central Norway. Ann. Neurol..

[2] Tomiyama H (2006). Clinicogenetic study of mutations in LRRK2 exon 41 in Parkinson’s disease patients from 18 countries. Mov Disord..

[3] Ishihara L (2006). Clinical features of Parkinson disease patients with homozygous leucine-rich repeat kinase 2 G2019S mutations. Arch. Neurol..

[4] Paisan-Ruiz C (2005). Familial Parkinson’s disease: clinical and genetic analysis of four Basque families. Ann. Neurol..

[5] Trinh, J., Farrer, M., Ross, O.A. & Guella, I. LRRK2-Related Parkinson Disease. GeneReviews® (2006).

[6] Mata IF (2012). Common variation in the LRRK2 gene is a risk factor for Parkinson’s disease. Mov Disord..

[7] Satake W (2009). Genome-wide association study identifies common variants at four loci as genetic risk factors for Parkinson’s disease. Nat. Genet..

[8] Skipper L (2005). Comprehensive evaluation of common genetic variation within LRRK2 reveals evidence for association with sporadic Parkinson’s disease. Hum. Mol. Genet..

[9] Ross OA (2011). Association of LRRK2 exonic variants with susceptibility to Parkinson’s disease: a case-control study. Lancet Neurol..

[10] Soto-Ortolaza AI (2013). GWAS risk factors in Parkinson’s disease: LRRK2 coding variation and genetic interaction with PARK16. Am. J. Neurodegener. Dis..

[11] Nalls MA (2011). Imputation of sequence variants for identification of genetic risks for Parkinson’s disease: a meta-analysis of genome-wide association studies. Lancet.

[12] Reyniers L (2014). Differential protein-protein interactions of LRRK1 and LRRK2 indicate roles in distinct cellular signaling pathways. J. Neurochem..

[13] Luerman GC (2014). Phosphoproteomic evaluation of pharmacological inhibition of leucine-rich repeat kinase 2 reveals significant off-target effects of LRRK-2-IN-1. J. Neurochem..

[14] Zach S, Felk S, Gillardon F (2010). Signal transduction protein array analysis links LRRK2 to Ste20 kinases and PKC zeta that modulate neuronal plasticity. PLoS. One..

[15] Zheng XY (2008). Screening of LRRK2 interactants by yeast 2-hybrid analysis. Zhong. Nan. Da. Xue. Xue. Bao. Yi. Xue. Ban..

[16] Meixner A (2011). A QUICK screen for Lrrk2 interaction partners–leucine-rich repeat kinase 2 is involved in actin cytoskeleton dynamics. Mol. Cell Proteomics..

[17] Mandemakers W, Snellinx A, O’Neill MJ, De SB (2012). LRRK2 expression is enriched in the striosomal compartment of mouse striatum. Neurobiol. Dis..

[18] Han BS (2008). Expression of the LRRK2 gene in the midbrain dopaminergic neurons of the substantia nigra. Neurosci. Lett..

[19] Miklossy J (2006). LRRK2 expression in normal and pathologic human brain and in human cell lines. J. Neuropathol. Exp. Neurol..

[20] Galter D (2006). LRRK2 expression linked to dopamine-innervated areas. Ann. Neurol..

[21] Simon-Sanchez J, Herranz-Perez V, Olucha-Bordonau F, Perez-Tur J (2006). LRRK2 is expressed in areas affected by Parkinson’s disease in the adult mouse brain. Eur. J. Neurosci..

[22] Westerlund M (2008). Developmental regulation of leucine-rich repeat kinase 1 and 2 expression in the brain and other rodent and human organs: Implications for Parkinson’s disease. Neuroscience.

[23] Heckman MG (2014). LRRK2 exonic variants and risk of multiple system atrophy. Neurology.

[24] Trabzuni D (2013). Fine-mapping, gene expression and splicing analysis of the disease associated LRRK2 locus. PLoS. One..

[25] Umeno J (2011). Meta-analysis of published studies identified eight additional common susceptibility loci for Crohn’s disease and ulcerative colitis. Inflamm. Bowel. Dis..

[26] Fava VM (2016). A Missense LRRK2 Variant Is a Risk Factor for Excessive Inflammatory Responses in Leprosy. PLoS. Negl. Trop. Dis..

[27] Wang D (2015). Association of the LRRK2 genetic polymorphisms with leprosy in Han Chinese from Southwest China. Genes Immun..

[28] Zhang FR (2009). Genomewide association study of leprosy. N. Engl. J. Med..

[29] Doggett EA, Zhao J, Mork CN, Hu D, Nichols RJ (2012). Phosphorylation of LRRK2 serines 955 and 973 is disrupted by Parkinson’s disease mutations and LRRK2 pharmacological inhibition. J. Neurochem..

[30] Dzamko N (2010). Inhibition of LRRK2 kinase activity leads to dephosphorylation of Ser(910)/Ser(935), disruption of 14-3-3 binding and altered cytoplasmic localization. Biochem. J..

[31] Dzamko N, Chua G, Ranola M, Rowe DB, Halliday GM (2013). Measurement of LRRK2 and Ser910/935 phosphorylated LRRK2 in peripheral blood mononuclear cells from idiopathic Parkinson’s disease patients. J. Parkinsons. Dis..

[32] Delbroek L (2013). Development of an enzyme-linked immunosorbent assay for detection of cellular and *in vivo* LRRK2 S935 phosphorylation. J. Pharm. Biomed. Anal..

[33] Deng X (2011). Characterization of a selective inhibitor of the Parkinson’s disease kinase LRRK2. Nat. Chem. Biol..

[34] Baptista MA (2013). Loss of leucine-rich repeat kinase 2 (LRRK2) in rats leads to progressive abnormal phenotypes in peripheral organs. PLoS. One..

[35] Herzig MC (2011). LRRK2 protein levels are determined by kinase function and are crucial for kidney and lung homeostasis in mice. Hum. Mol. Genet..

[36] Tong Y (2010). Loss of leucine-rich repeat kinase 2 causes impairment of protein degradation pathways, accumulation of alpha-synuclein, and apoptotic cell death in aged mice. Proc. Natl. Acad. Sci. USA.

[37] Fell MJ (2015). MLi-2, a Potent, Selective, and Centrally Active Compound for Exploring the Therapeutic Potential and Safety of LRRK2 Kinase Inhibition. J. Pharmacol. Exp. Ther..

[38] Fuji RN (2015). Effect of selective LRRK2 kinase inhibition on nonhuman primate lung. Sci. Transl. Med..

[39] Sheng Z (2012). Ser1292 autophosphorylation is an indicator of LRRK2 kinase activity and contributes to the cellular effects of PD mutations. . Sci. Transl. Med..

[40] Reynolds A, Doggett EA, Riddle SM, Lebakken CS, Nichols RJ (2014). LRRK2 kinase activity and biology are not uniformly predicted by its autophosphorylation and cellular phosphorylation site status. Front Mol. Neurosci..

[41] Steger, M. *et al*. Phosphoproteomics reveals that Parkinson’s disease kinase LRRK2 regulates a subset of Rab GTPases. *Elife*. 5 (2016). 10.7554/eLife.12813 PMC4769169 26824392

[42] Tan EK (2010). Analysis of GWAS-linked loci in Parkinson disease reaffirms PARK16 as a susceptibility locus. Neurology.

[43] Fraser KB, Moehle MS, Alcalay RN, West AB (2016). Urinary LRRK2 phosphorylation predicts parkinsonian phenotypes in G2019S LRRK2 carriers. Neurology.

[44] Fraser, K.B. *et al*. Ser(P)-1292 LRRK2 in urinary exosomes is elevated in idiopathic Parkinson’s disease. *Mov Disord* (2016). 10.1002/mds.26686 PMC5053851 27297049

[45] Fraser KB (2013). LRRK2 secretion in exosomes is regulated by 14-3-3. Hum. Mol. Genet..

[46] Henderson JL (2015). Discovery and preclinical profiling of 3-[4-(morpholin-4-yl)-7H-pyrrolo[2,3-d]pyrimidin-5-yl]benzonitrile (PF-06447475), a highly potent, selective, brain penetrant, and *in vivo* active LRRK2 kinase inhibitor. J. Med. Chem..

[47] Hatcher JM (2015). Discovery of a Pyrrolopyrimidine (JH-II-127), a Highly Potent, Selective, and Brain Penetrant LRRK2 Inhibitor. ACS Med. Chem. Lett..

[48] Reith AD (2012). GSK2578215A; a potent and highly selective 2-arylmethyloxy-5-substitutent-N-arylbenzamide LRRK2 kinase inhibitor. Bioorg. Med. Chem. Lett..

[49] Zhang J, Deng X, Choi HG, Alessi DR, Gray NS (2012). Characterization of TAE684 as a potent LRRK2 kinase inhibitor. Bioorg. Med. Chem. Lett..

[50] Estrada AA (2014). Discovery of highly potent, selective, and brain-penetrant aminopyrazole leucine-rich repeat kinase 2 (LRRK2) small molecule inhibitors. J. Med. Chem..

[51] Thevenet J, Pescini GR, Hooft van HR, Wiessner C, Sagot YJ (2011). Regulation of LRRK2 expression points to a functional role in human monocyte maturation. PLoS. One..

[52] Kubo M (2010). LRRK2 is expressed in B-2 but not in B-1 B cells, and downregulated by cellular activation. J. Neuroimmunol..

[53] Hakimi M (2011). Parkinson’s disease-linked LRRK2 is expressed in circulating and tissue immune cells and upregulated following recognition of microbial structures. J. Neural Transm. (Vienna.).

[54] Gardet A (2010). LRRK2 is involved in the IFN-gamma response and host response to pathogens. J. Immunol..

[55] Bedford, S. T., Chen, I.-J., Wang, Y. & Williamson, D. S. Preparation of 4,6-disubstituted 1*H*-pyrrolo[2,3-*b*]pyridine-3-carbonitrile compounds as LRRK2 inhibitors. [WO 2014170248]. 2014. Ref Type: Patent.

[56] Nichols RJ (2009). Substrate specificity and inhibitors of LRRK2, a protein kinase mutated in Parkinson’s disease. Biochem. J..

[57] Lobbestael E (2016). Pharmacological LRRK2 kinase inhibition induces LRRK2 protein destabilization and proteasomal degradation. Sci. Rep..

[58] Zhao J, Molitor TP, Langston JW, Nichols RJ (2015). LRRK2 dephosphorylation increases its ubiquitination. Biochem. J..

[59] Ito G (2016). Phos-tag analysis of Rab10 phosphorylation by LRRK2: a powerful assay for assessing kinase function and inhibitors. Biochem. J..

[60] Kuwahara T (2016). LRRK2 and RAB7L1 coordinately regulate axonal morphology and lysosome integrity in diverse cellular contexts. Sci. Rep..

[61] Yun HJ (2015). An early endosome regulator, Rab5b, is an LRRK2 kinase substrate. J. Biochem..

[62] Waschbusch D (2014). LRRK2 transport is regulated by its novel interacting partner Rab32. PLoS. One..

[63] Gomez-Suaga P (2014). LRRK2 delays degradative receptor trafficking by impeding late endosomal budding through decreasing Rab7 activity. Hum. Mol. Genet..

[64] Martin I (2014). Ribosomal protein s15 phosphorylation mediates LRRK2 neurodegeneration in Parkinson’s disease. Cell.

[65] MacLeod DA (2013). RAB7L1 interacts with LRRK2 to modify intraneuronal protein sorting and Parkinson’s disease risk. Neuron.

[66] Shin N (2008). LRRK2 regulates synaptic vesicle endocytosis. Exp. Cell Res..

[67] Nikonova EV (2012). Transcriptional responses to loss or gain of function of the leucine-rich repeat kinase 2 (LRRK2) gene uncover biological processes modulated by LRRK2 activity. Hum. Mol. Genet..

[68] Ito G, Fujimoto T, Kamikawaji S, Kuwahara T, Iwatsubo T (2014). Lack of correlation between the kinase activity of LRRK2 harboring kinase-modifying mutations and its phosphorylation at Ser910, 935, and Ser955. PLoS. One..

[69] Trancikova A (2012). Phosphorylation of 4E-BP1 in the mammalian brain is not altered by LRRK2 expression or pathogenic mutations. PLoS. One..

[70] Liu Z (2011). The kinase LRRK2 is a regulator of the transcription factor NFAT that modulates the severity of inflammatory bowel disease. Nat. Immunol..

[71] Gehrke S, Imai Y, Sokol N, Lu B (2010). Pathogenic LRRK2 negatively regulates microRNA-mediated translational repression. Nature.

[72] Hermanson SB (2012). Screening for novel LRRK2 inhibitors using a high-throughput TR-FRET cellular assay for LRRK2 Ser935 phosphorylation. PLoS. One..

[73] Pfeffer SR (2005). Structural clues to Rab GTPase functional diversity. J. Biol. Chem..

[74] Baptista, M.A.S. *et al*. LRRK2 Kinase Inhibitors of Different Structural Classes Induce Abnormal Accumulation of Lamellar Bodies in Type II Pneumocytes in Non-Human Primates but are Reversible and Without Pulmonary Functional Consequences. *Michael J*. *Fox Foundation*. (2015) Ref Type: Online Source.

[75] Stenmark H (2009). Rab GTPases as coordinators of vesicle traffic. Nat. Rev. Mol. Cell Biol..

[76] Beilina A (2014). Unbiased screen for interactors of leucine-rich repeat kinase 2 supports a common pathway for sporadic and familial Parkinson disease. Proc. Natl. Acad. Sci. USA.

[77] Wang L (2017). Evaluation of the interaction between LRRK2 and PARK16 loci in determining risk of Parkinson’s disease: analysis of a large multicenter study. Neurobiol. Aging.

[78] Lai YC (2015). Phosphoproteomic screening identifies Rab GTPases as novel downstream targets of PINK1. EMBO J.

[79] Yin G (2014). alpha-Synuclein interacts with the switch region of Rab8a in a Ser129 phosphorylation-dependent manner. Neurobiol. Dis..

[80] Goncalves SA (2016). shRNA-Based Screen Identifies Endocytic Recycling Pathway Components That Act as Genetic Modifiers of Alpha-Synuclein Aggregation, Secretion and Toxicity. PLoS. Genet..

[81] Wilson GR (2014). Mutations in RAB39B cause X-linked intellectual disability and early-onset Parkinson disease with alpha-synuclein pathology. Am. J Hum. Genet..

[82] Chutna O (2014). The small GTPase Rab11 co-localizes with alpha-synuclein in intracellular inclusions and modulates its aggregation, secretion and toxicity. Hum. Mol. Genet..

[83] Soper JH, Kehm V, Burd CG, Bankaitis VA, Lee VM (2011). Aggregation of alpha-synuclein in S. cerevisiae is associated with defects in endosomal trafficking and phospholipid biosynthesis. J Mol. Neurosci..

[84] Liu J (2009). Rab11a and HSP90 regulate recycling of extracellular alpha-synuclein. J Neurosci..

[85] Gitler AD (2008). The Parkinson’s disease protein alpha-synuclein disrupts cellular Rab homeostasis. Proc. Natl. Acad. Sci. USA.

[86] Dalfo E (2004). Abnormal alpha-synuclein interactions with Rab proteins in alpha-synuclein A30P transgenic mice. J Neuropathol. Exp. Neurol..

[87] Dalfo E, Barrachina M, Rosa JL, Ambrosio S, Ferrer I (2004). Abnormal alpha-synuclein interactions with rab3a and rabphilin in diffuse Lewy body disease. Neurobiol. Dis..

[88] Elferink LA, Anzai K, Scheller RH (1992). rab15, a novel low molecular weight GTP-binding protein specifically expressed in rat brain. J Biol. Chem..

[89] Chen Y, Lippincott-Schwartz J (2013). Rab10 delivers GLUT4 storage vesicles to the plasma membrane. Commun. Integr. Biol..

[90] Sano H, Roach WG, Peck GR, Fukuda M, Lienhard GE (2008). Rab10 in insulin-stimulated GLUT4 translocation. Biochem. J.

[91] Sano H (2007). Rab10, a target of the AS160 Rab GAP, is required for insulin-stimulated translocation of GLUT4 to the adipocyte plasma membrane. Cell Metab.

[92] Deen AJ (2014). Rab10-mediated endocytosis of the hyaluronan synthase HAS3 regulates hyaluronan synthesis and cell adhesion to collagen. J Biol. Chem..

[93] Wang D (2010). Ras-related protein Rab10 facilitates TLR4 signaling by promoting replenishment of TLR4 onto the plasma membrane. Proc. Natl. Acad. Sci. USA.

[94] Schuck S (2007). Rab10 is involved in basolateral transport in polarized Madin-Darby canine kidney cells. Traffic..

[95] Babbey CM (2006). Rab10 regulates membrane transport through early endosomes of polarized Madin-Darby canine kidney cells. Mol. Biol. Cell.

[96] Li Z (2016). A novel Rab10-EHBP1-EHD2 complex essential for the autophagic engulfment of lipid droplets. Sci. Adv..

[97] Ness D (2013). Leucine-rich repeat kinase 2 (LRRK2)-deficient rats exhibit renal tubule injury and perturbations in metabolic and immunological homeostasis. PLoS. One..

[98] Wang Y (2011). Reversed-phase chromatography with multiple fraction concatenation strategy for proteome profiling of human MCF10A cells. Proteomics..

[99] Mertins P (2013). Integrated proteomic analysis of post-translational modifications by serial enrichment. Nat. Methods.

[100] Rappsilber J, Mann M, Ishihama Y (2007). Protocol for micro-purification, enrichment, pre-fractionation and storage of peptides for proteomics using StageTips. Nat. Protoc..

[101] Cox J, Mann M (2008). MaxQuant enables high peptide identification rates, individualized p.p.b.-range mass accuracies and proteome-wide protein quantification. Nat. Biotechnol..

[102] Olsen JV (2006). Global, *in vivo*, and site-specific phosphorylation dynamics in signaling networks. Cell.

[103] Klammer M, Dybowski JN, Hoffmann D, Schaab C (2014). Identification of significant features by the Global Mean Rank test. PLoS. One..

[104] Szklarczyk D (2015). STRING v10: protein-protein interaction networks, integrated over the tree of life. Nucleic Acids Res..

[105] Afsari F (2014). Abnormal visual gain control in a Parkinson’s disease model. Hum. Mol. Genet..

