# Supplementary material for: Selective LRRK2 kinase inhibition reduces phosphorylation of endogenous Rab10 and Rab12 in human peripheral mononuclear blood cells
Source: Sci Rep. 2017 Aug 31;7:10300. doi: 10.1038/s41598-017-10501-z (PMC5578959; doi:10.1038/s41598-017-10501-z)
Supplement: Supplementary file 1 — Supplementary Data File [file 41598_2017_10501_MOESM1_ESM.pdf]

Supplementary Dataset File

Selective LRRK2 kinase inhibition reduces phosphorylation of endogenous Rab10 and Rab12 in human peripheral mononuclear blood cells

Kenneth Thirstrup[1], Justus C. Dächsel[1], Felix S. Oppermann[4], Douglas S. Williamson[3], Garrick P. Smith[2], Karina Fog [1] and Kenneth Vielsted Christensen[1†]

H. Lundbeck A/S, [1]Neurodegeneration and Biologics, [2]Discovery Chemistry, Ottiliavej 9, DK-2500 Valby, Denmark;[3]Vernalis (R&D) Ltd, Granta Park, Great Abington, Cambridge, CB21 6GB, United Kingdom;[4]Evotec (Münich) GmbH, Am Klopferspitz 19a, 82152 Martinsried, Germany

Supplementary Table 1

Replicate experiment	mTRAQ labeling efficiency Δ0 (light) [%]	mTRAQ labeling efficiency Δ4 (medium) [%]	mTRAQ labeling efficiency Δ8 (heavy) [%]
1	99,7	99,0	98,3
2	99,7	98,9	98,9
3	99,7	98,9	98,9
4	99,7	99,1	99,0
5	99,7	98,9	98,9
6	99,7	99,3	99,0
7	99,7	99,1	99,1
8	99,7	99,3	98,8

Supplementary Table 2

Sample	Dilution from 1mg/ml	Antibody concentration (mg/ml)	Rabbit anti-human Rab10-pThr73 antibody (#5781)	
			Rab10-pThr73 peptide	Rab10 peptide
NC	1:1,000	N/A	0.073	0.077
1	1:1,000	1,000.00	3.511	0.107
2	1:2,000	500.00	3.371	0.086
3	1:4,000	250.00	3.283	0.078
4	1:8,000	125.00	3.054	0.071
5	1:16,000	62.50	2.818	0.069
6	1:32,000	31.25	2.478	0.066
7	1:64,000	15.62	2.018	0.064
8	1:128,000	7.81	1.390	0.064
9	1:256,000	3.90	0.869	0.062
10	1:512,000	1.95	0.464	0.062
11	Blank	Blank	0.062	0.086
12	Blank	Blank	0.062	0.086

Supplementary Table 3

Sample	Dilution from 1mg/ml	Antibody concentration (mg/ml)	Rabbit anti-human Rab12-pSer106 antibody (#5919)	
			Rab12-pSer106 peptide	Rab12 peptide
NC	1:1,000	N/A	0.061	0.052
1	1:1,000	1,000.00	3.249	0.138
2	1:2,000	500.00	3.227	0.128
3	1:4,000	250.00	3.047	0.102
4	1:8,000	125.00	2.637	0.084
5	1:16,000	62.50	2.406	0.078
6	1:32,000	31.25	1.832	0.070
7	1:64,000	15.62	1.165	0.068
8	1:128,000	7.81	0.608	0.065
9	1:256,000	3.90	0.354	0.064
10	1:512,000	1.95	0.206	0.061
11	Blank	Blank	0.057	0.074
12	Blank	Blank	0.057	0.074

Supplementary Table 4

Protein ID (UniProtKB)	Gene Name	p-site	p-site ratio (CMPD/DMSO)	Protein ratio (CMPD/DMSO)	q-value Mean Rank CMPD/DMSO	No. of replicates with ratio
Q5S007	LRRK2	Ser850	1,04	0,6	n.a.	2
Q5S007	LRRK2	**Ser910**	**0,19**	**0,6**	**0.000**	**8**
Q5S007	LRRK2	Ser912	0,84	0,6	0,998	7
Q5S007	LRRK2	Ser933	0,11	0,6	n.a.	3
Q5S007	LRRK2	**Ser935**	**0,11**	**0,6**	**0.000**	**8**
Q5S007	LRRK2	**Ser955**	**0,41**	**0,6**	**0,005**	**5**
Q5S007	LRRK2	Ser958	1,3	0,6	n.a.	2
Q5S007	LRRK2	**Ser973**	**0,39**	**0,6**	**0,003**	**8**

Supplementary Table 5

Protein ID (UniProtKB)	Gene Name	Similar phosphopeptide (Gene Name)	p-site	p-site ratio	Protein ratio	q-value	No. of p-sites replicates	No. of protein replicates
Q6IQ22	**RAB12**	unique	**Ser106**	**0,42**	1.30	**0,000**	**5**	7
P61026	**RAB10**	unique	**Thr73**	**0,57**	1.10	**0,008**	**8**	8
P61026	RAB10	unique	Thr75	0,98	1.10	0,953	5	8
Q9H0U4	RAB1A	RAB1B	Ser75	1,07	0.99	0,953	6	8
O00194	RAB27A	RAB27B	Ser83	1,02	0.93	0,953	8	8
Q15907	RAB11B	RAB11A; RAB25	Ser78	1,03	1.09	0,953	8	8
Q9NRW1	RAB6A	RAB6B	Ser77	1,02	1.07	0,953	8	8
Q9H0U4	RAB1A	RAB1B	Thr72	1,00	0.99	0,953	8	8
P61019	RAB2A	RAB2B	Ser70	0,98	1.04	0,953	6	8
Q5U602	RAB33B	unique	Ser98	0,95	1.00	0,953	5	7
P51149	RAB7A	unique	Ser72	0,99	1.00	0,954	8	8
P61006	RAB8A	RAB8B	Thr72	0,86	1.07	0,998	8	8

Supplementary Figure 1

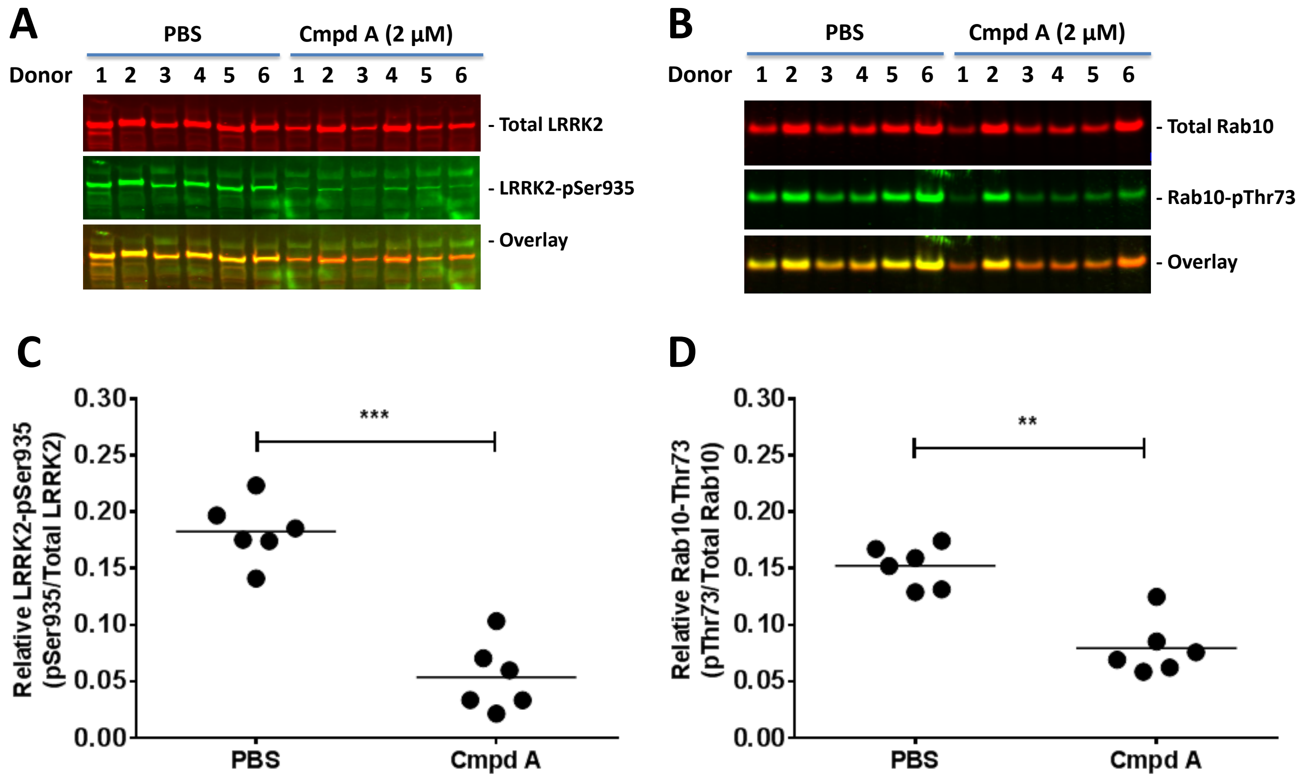

Supplementary Figure 1. Acute LRRK2 inhibition with Compound A (Cmpd A) reduces LRRK2-pSer935 and Rab10-Thr73 phosphorylation in non-stimulated PBMCs from human healthy subjects. LiCor Western Blot images showing ***A***, LRRK2 and ***B***, Rab10 immunoreactivity (red panels), LRRK2-pSer935 and Rab10-pThr73 immunoreactivity (green panels) as well as overlay in non-stimulated human PBMCs treated with 2µM Cmpd A. Full-length blots are presented in Supplementary Figure 8. Quantification of ***C***, relative LRRK2-pSer935/total LRRK2 ratio and ***D***, relative Rab10-pThr73/total Rab10 ratio (n=6 donors; 2 conditions). Data was analyzed by paired t-test. Data is presented as means ± SEM; ***p<0.001 and **p<0.01 vs. PBS.

Supplementary Figure 2

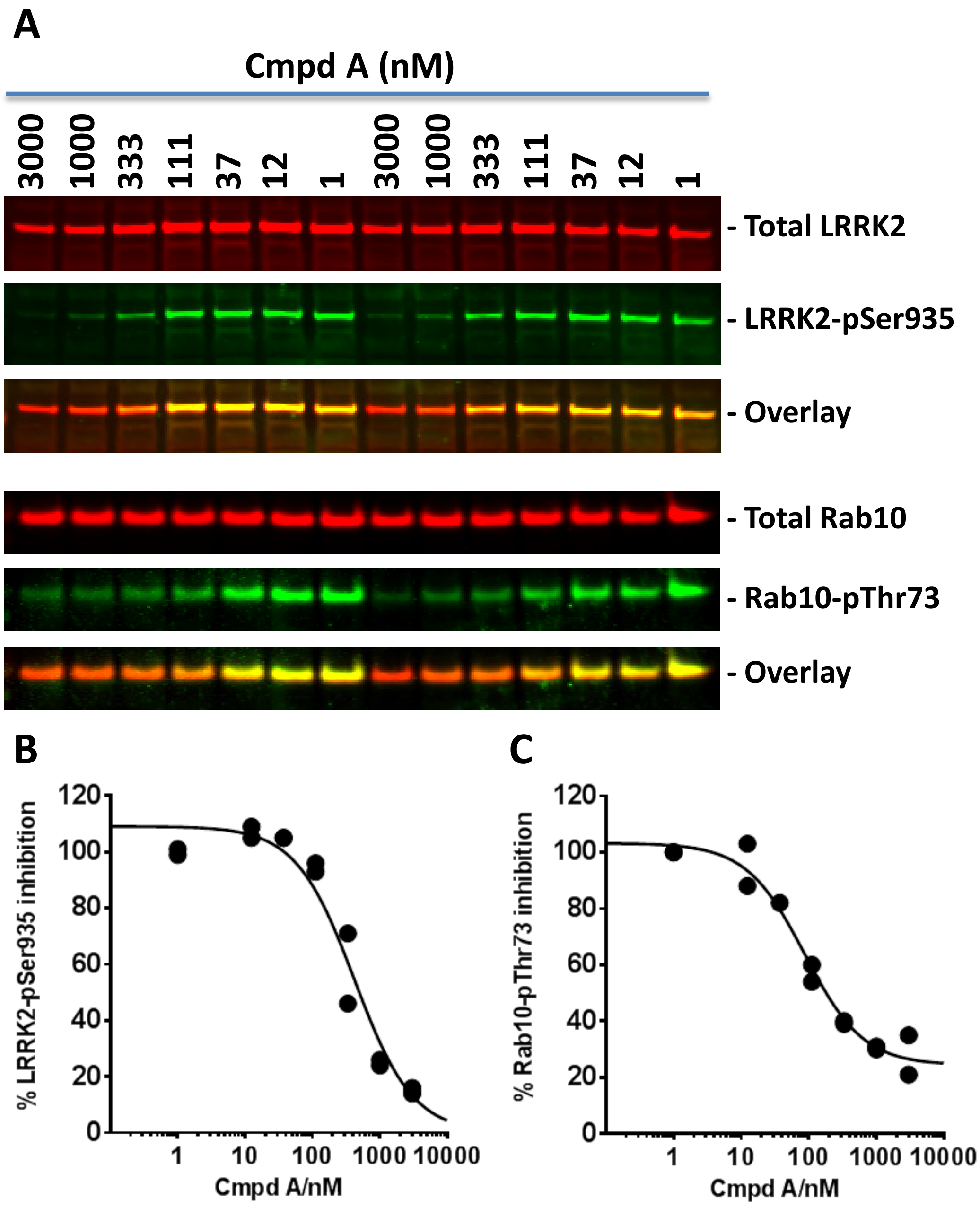

Supplementary Figure 2. Compound A (Cmpd A) inhibits LRRK2-Ser935 and Rab10-Thr73 phosphorylation in a concentration-dependent manner in non-stimulated PBMCs from human healthy subjects. Determination of LRRK2 inhibitor IC_{50} values based on either Rab10-pThr73 or LRRK2-pSer935 levels in human non-stimulated PBMCs. ***A***, LiCor image showing Western Blot analysis of crude lysates from a pool of PBMCs from two donors treated for 1 hour with concentrations of Cmpd A ranging from 1nM-3μM. in duplicate. LRRK2 and Rab10 immunoreactivity (*red panels*), LRRK2-pSer935 and Rab10-pThr73 immunoreactivity (*green panels*) as well as overlay in non-stimulated human PBMCs. Full-length blots are presented in Supplementary Figure 9. Non-linear regression plot of percentage ***B***, LRRK2-pSer935 inhibition and ***C***, Rab10-pThr73 as a function of log10-transformed Cmpd A concentration. The experiment was repeated three times and the resulting IC50 determination is summarized in Table 2.

Supplementary Figure 3

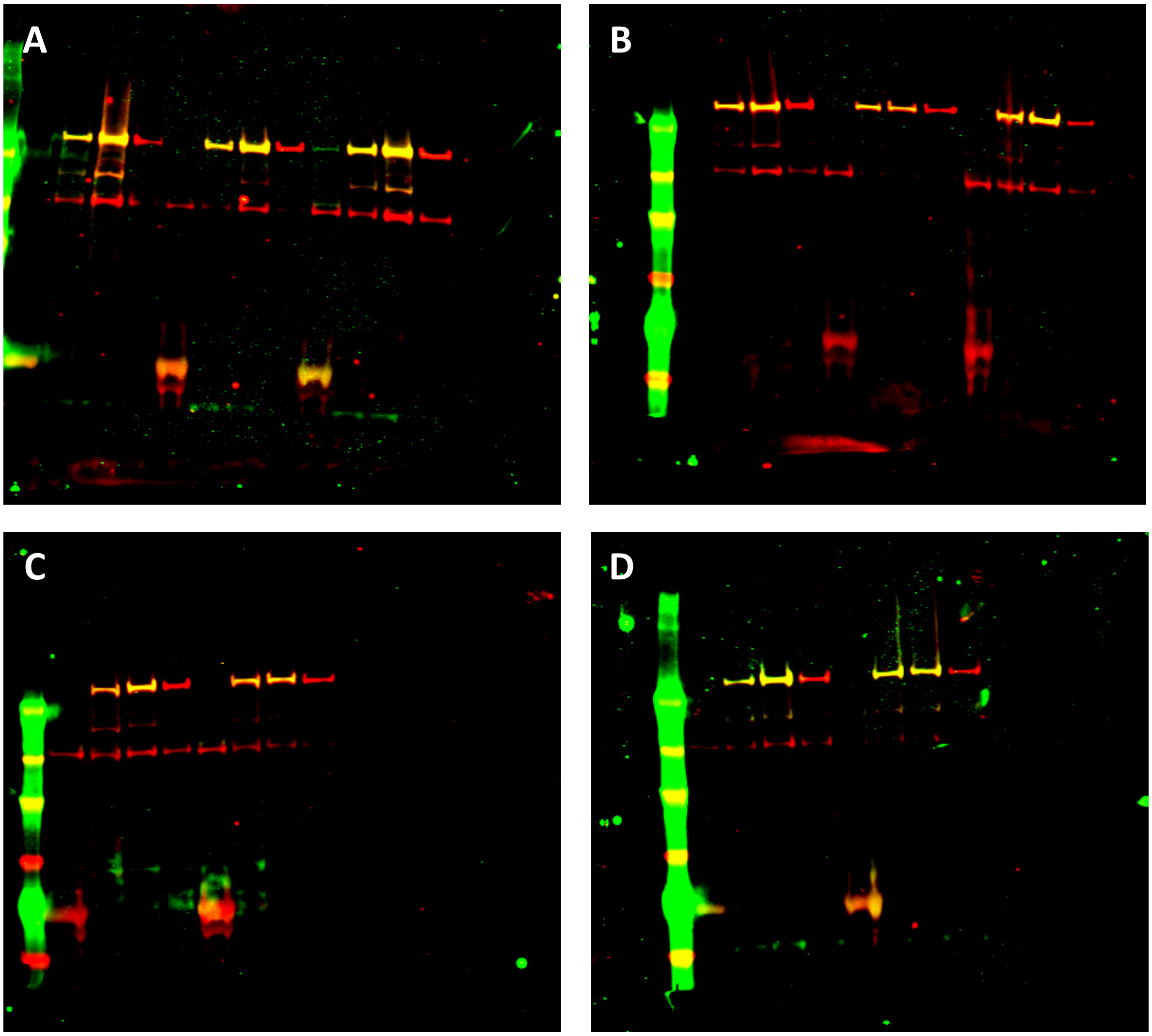

Supplementary Figure 3. *A-D*, Uncropped and unprocessed LiCor-CXL images of Western Blot used for estimation of LRRK2 protein levels (total LRRK2; red) and LRRK2 phosphorylation (pSer935; green) in Figure 1. *A*, donor 1-3; *B*, donor 4-6; *C*, donor 7-8; *D*, donor 9-10. Samples were loaded as follows: Lane 1, MW marker; lane 2, uncultured PBMCs; lane 3, cultured PBMCs; lane 4, cultured, stimulated and DMSO-treated PBMCs; lane 5, cultured, stimulated and Lu AF58786-treated PBMCs. Isolation of hPBMCs using the Leucosep procedures did not yield high levels of LRRK2 expression in the uncultured condition. Thus, for the sake of clarity the quantification of the uncultured hPBMCs have been omitted from Figure 1.

Supplementary Figure 4

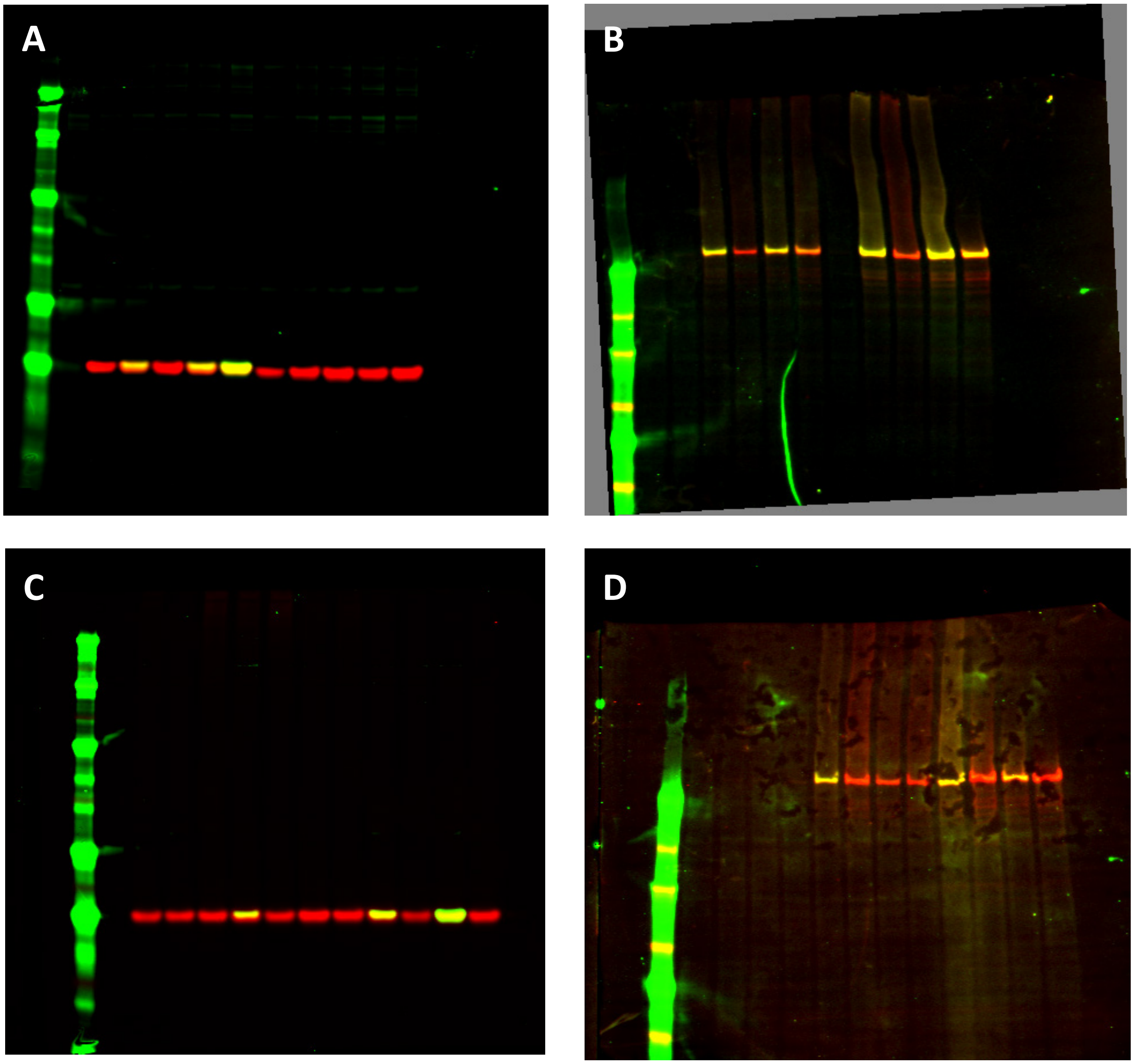

Supplementary Figure 4. *A-D*, Uncropped and unprocessed LiCor Western Blot images used in Figure 5 showing overexpressed total LRRK2 and HA-tagged Rab10 immunoreactivity (red), LRRK2-pSer935 and Rab10-pThr73 immunoreactivity (green) as well as overlay in cells overexpressing various LRRK2 exonic variants together with either HA-tagged wild type Rab10 or HA-tagged phosphodeficient Rab10-T73V. A-C, Rab10 and Rab10-pThr73. B-D, LRRK2 and LRRK2-pSer935. Samples were loaded as indicated in Figure 5.

Supplementary Figure 5

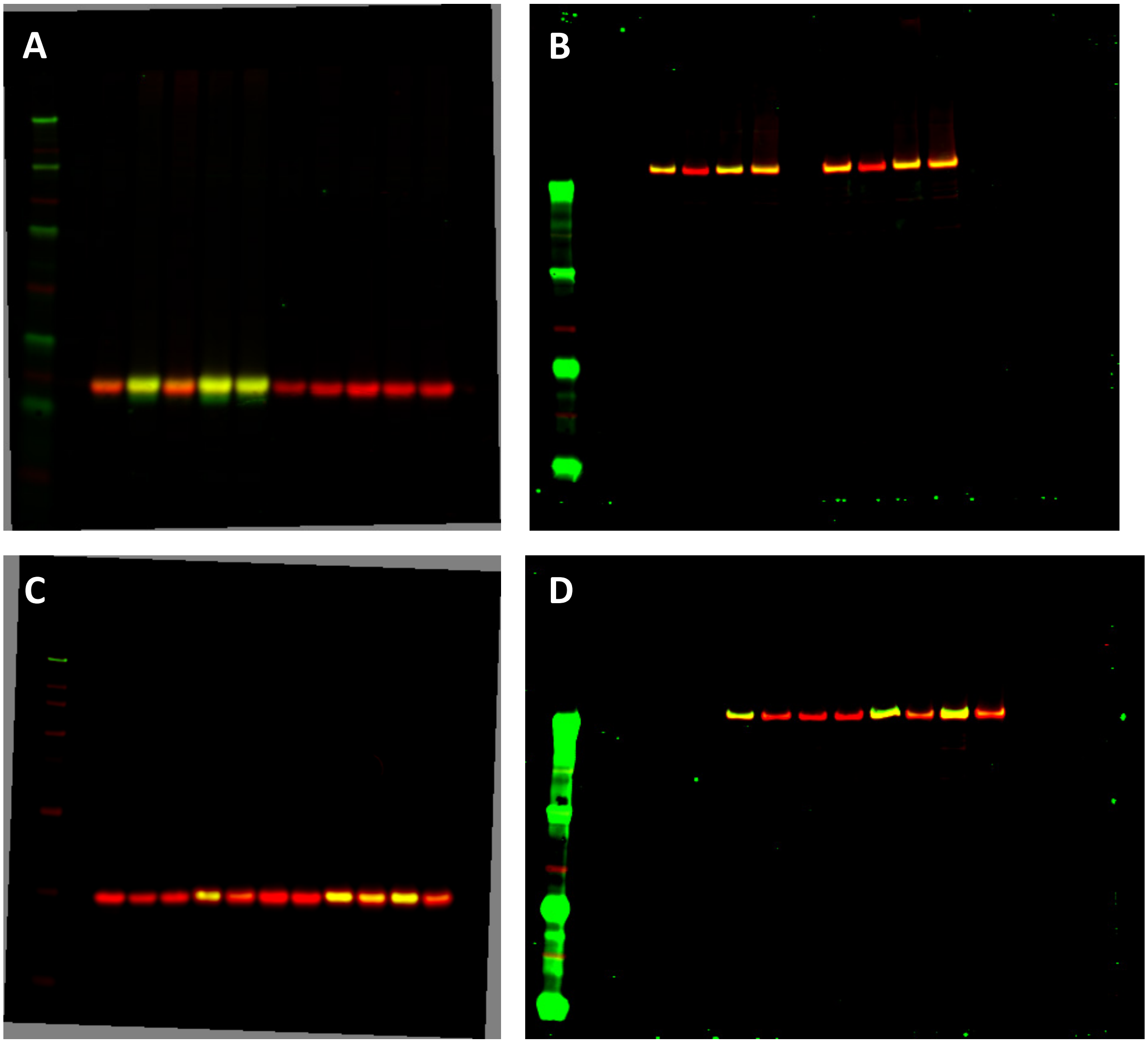

Supplementary Figure 5. *A-D*, Uncropped and unprocessed LiCor Western Blot images used in Figure 6 that shows overexpressed total LRRK2 and HA-tagged Rab12 immunoreactivity (red), LRRK2-pSer935 and Rab12-pSer106 immunoreactivity (green) as well as overlay in cells overexpressing various LRRK2 exonic variants together with either HA-tagged wild type Rab12 or HA-tagged phosphodeficient Rab12-S106A. A-C, Rab12 and Rab12-pSer106. B-D, LRRK2 and LRRK2-pSer935. Samples were loaded as indicated in Figure 6.

Supplementary Figure 6

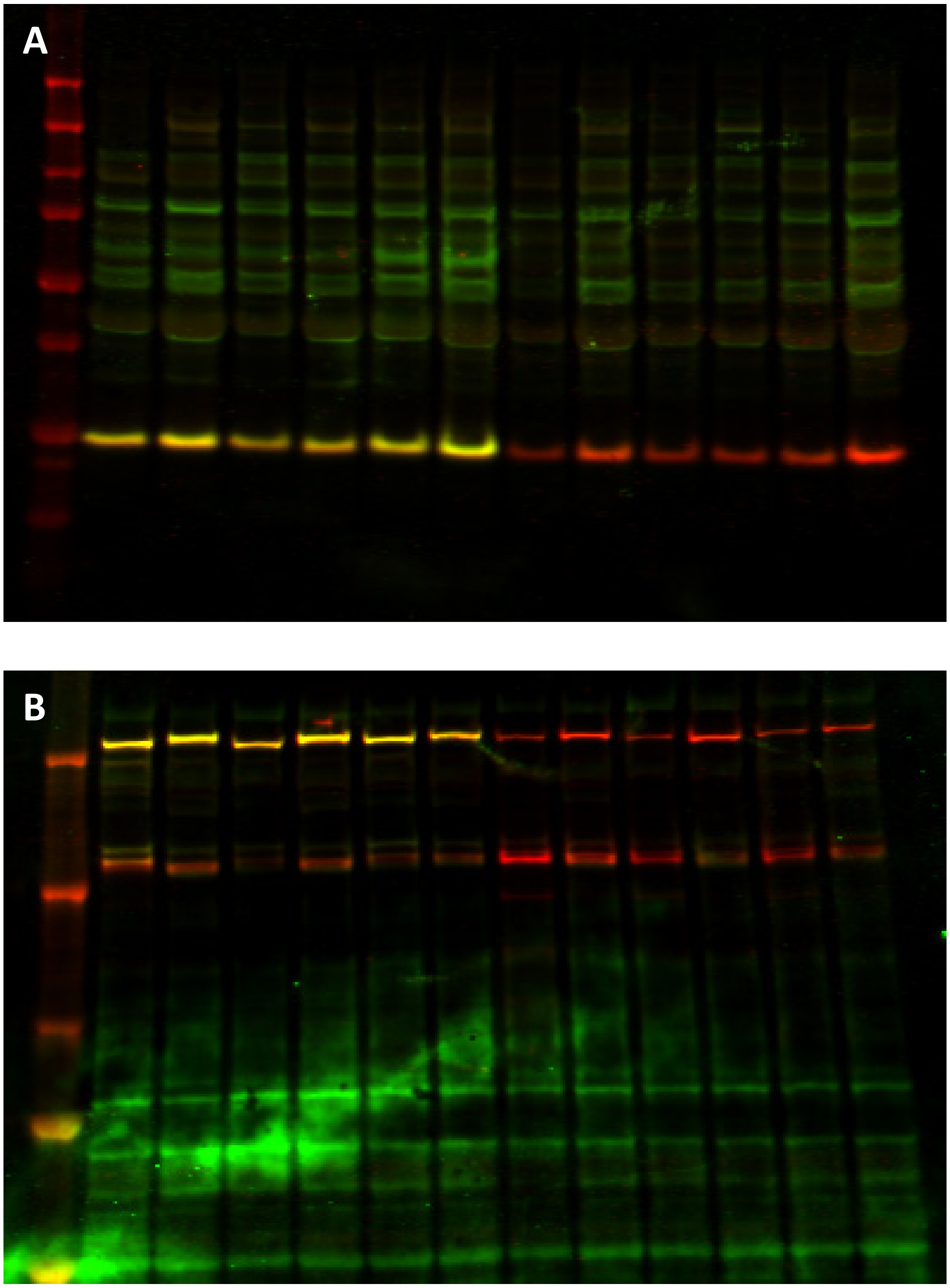

Supplementary Figure 6. Uncropped and unprocessed LiCor Western Blot images from figure 8 showing *A*, overlay of Rab10 (red) and Rab10-pThr73 immunoreactivity (green); *B*, overlay of LRRK2 (red) and LRRK2-pSer935 immunoreactivity (green) in Lymphoprep™ isolated and non-stimulated human PBMCs treated with 2μM PFE-360. Samples were loaded as indicated in Figure 8.

Supplementary Figure 7

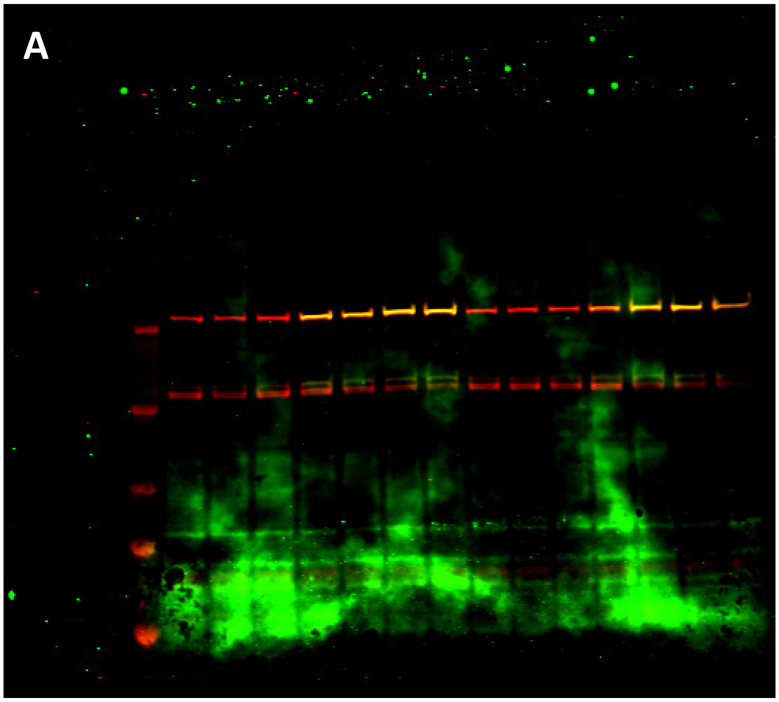

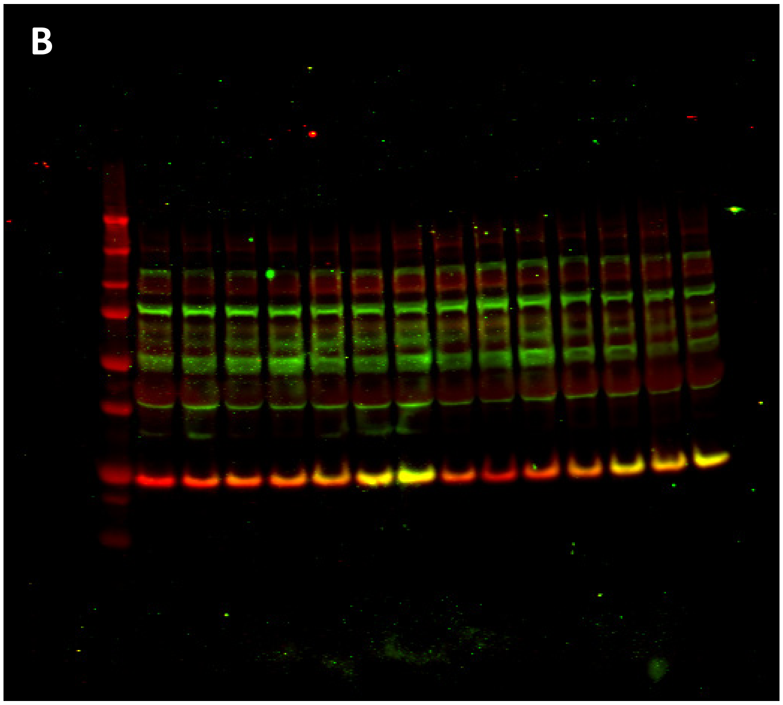

Supplementary Figure 7. Uncropped and unprocessed LiCor image from Figure 9 showing Western Blot analysis of crude lysates from a pool of PBMCs from two donors treated for 1 hour with concentrations of PFE-360 ranging from 4nM-1µM. in duplicate determination. *A*, overlay of Rab10 (red) and Rab10-pThr73 immunoreactivity (green); *B*, overlay of LRRK2 (red) and LRRK2-pSer935 immunoreactivity (green) in Lymphoprep™ isolated and non-stimulated human PBMCs. Samples were loaded as indicated in Figure 9.

Supplementary Figure 8

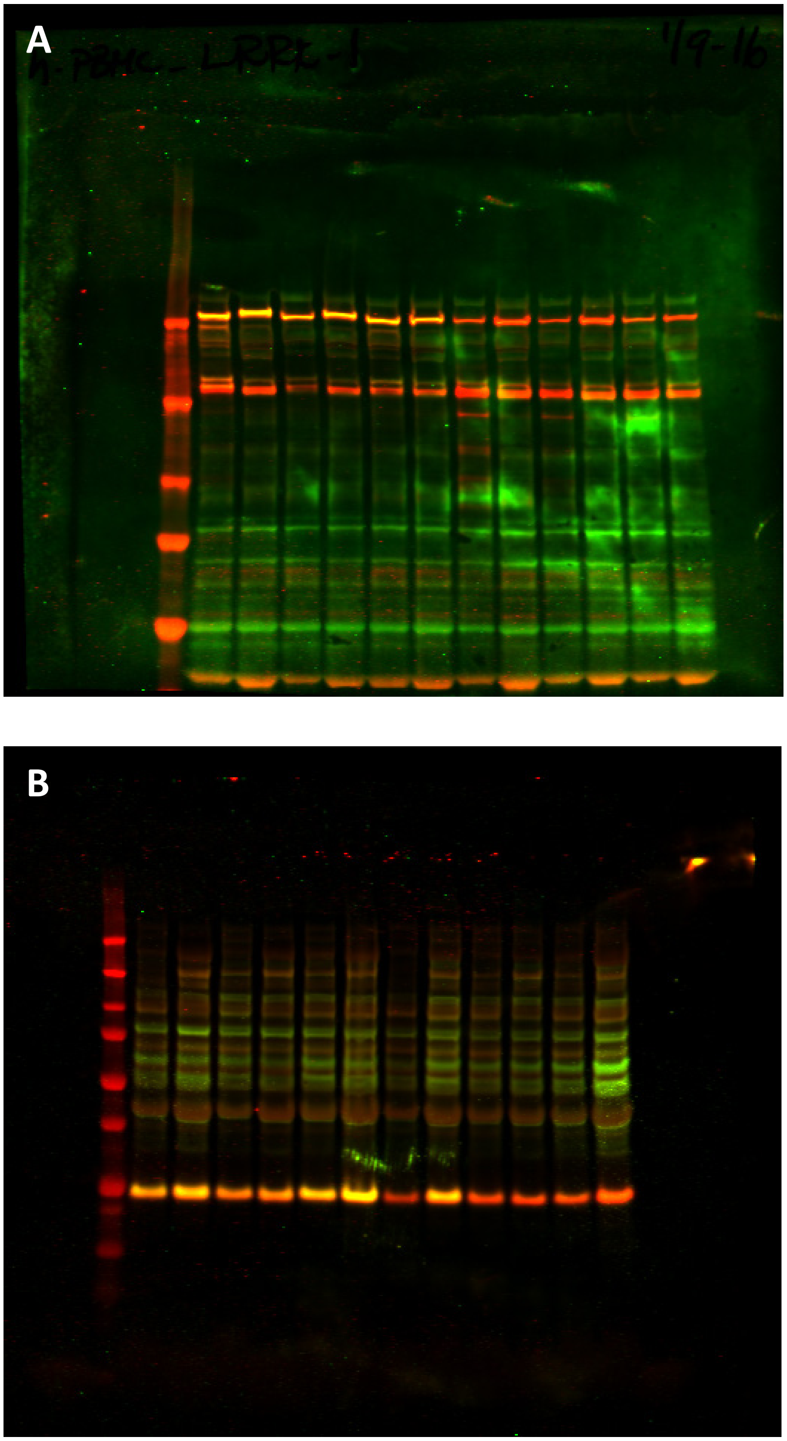

Supplementary Figure 8. Uncropped and unprocessed LiCor Western Blot images from Supplementary Figure 1 showing *A*, overlay of Rab10 (red) and Rab10-pThr73 immunoreactivity (green); *B*, overlay of LRRK2 (red) and LRRK2-pSer935 immunoreactivity (green) in Lymphoprep™ isolated and non-stimulated human PBMCs treated with 2μM Cmpd A. Samples were loaded as indicated in Supplementary Figure 1.

Supplementary Figure 9

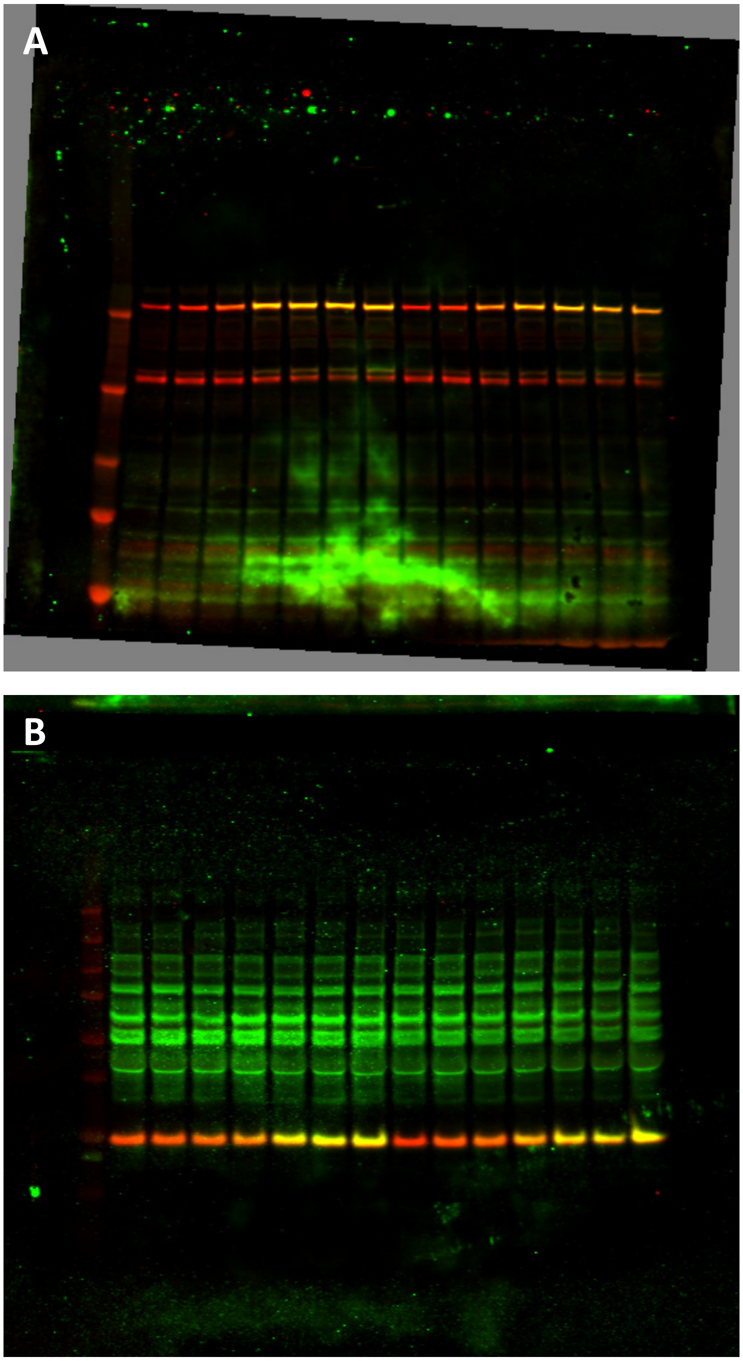

Supplementary Figure 9. Uncropped and unprocessed LiCor image from Supplementary Figure 2 showing Western Blot analysis of crude lysates from a pool of PBMCs from two donors treated for 1 hour with concentrations of Cmpd A ranging from 1nM-3µM. in duplicate determination. *A*, overlay of Rab10 (red) and Rab10-pThr73 immunoreactivity (green); *B*, overlay of LRRK2 (red) and LRRK2-pSer935 immunoreactivity (green) in Lymphoprep™ isolated and non-stimulated human PBMCs. Samples were loaded as indicated in Supplementary Figure 2.
